# Antimicrobial Resistance Profiles, Resistance Genes, and Their Association with Bacteriophage Susceptibility Among *Vibrio parahaemolyticus* Isolates from Zhanjiang Shrimp Aquaculture Farms

**DOI:** 10.3390/microorganisms14092004

**Published:** 2026-09-09

**Authors:** Lukman Iddrisu, Baochen Chai, Evodia Moses Mkulo, Salifu Ibrahim, Felix Danso, Muqadas Altaf, Muhammad Fahad Khan, Bingyu Zhang, Shumei Zhang, Jiesen Su, Yinyan Chen, Zhijia Fang, Jianzhi Ye

**Affiliations:** 1 Guangdong Provincial Key Laboratory of Aquatic Product Processing and Safety, Guangdong Provincial Engineering Technology, Research Center of Marine Food, Key Laboratory of Advanced Processing of Aquatic Products of Guangdong Higher Education Institution, College of Food Science and Technology, Guangdong Ocean University, Zhanjiang 524088, China; lukmaniddrisu54@gmail.com (L.I.); muqadasaltaf304@gmail.com (M.A.); fahadkhan31202@gmail.com (M.F.K.); m19513320507@163.com (B.Z.); zhangshumei0504@163.com (S.Z.); 15813276882@163.com (J.S.); rqxchenyinyan@163.com (Y.C.); 2 Food Quality Testing Center of Ministry of Agriculture and Rural Affairs, Agricultural Products Processing Research Institute, Chinese Academy of Tropical Agricultural Sciences, Zhanjiang 524001, China; 3 Licensing Inspection Center of Shenzhen Administration for Market Regulation, Shenzhen 518024, China; cbc973@126.com; 4 Guangdong Provincial Key Laboratory of Pathogenic Biology and Epidemiology for Aquatic Economic Animals, College of Fisheries, Guangdong Ocean University, Zhanjiang 524088, China; evomkulo@gmail.com (E.M.M.); ibsuala11@gmail.com (S.I.); 5 Department of Veterinary Medicine, Guangdong Ocean University, Zhanjiang 524088, China; fdanfreeman@gmail.com

**Keywords:** *Vibrio parahaemolyticus*, antimicrobial resistance, multidrug resistance, bacteriophage susceptibility, antibiotic resistance genes

## Abstract

*Vibrio parahaemolyticus* is an important foodborne and aquaculture-associated pathogen, and its increasing antimicrobial resistance threatens shrimp production, seafood safety, and public health. Although bacteriophages have emerged as a potential solution to the growing challenge of antimicrobial resistance, whether antibiotic resistance is associated with phage tolerance remains unclear. This study characterized antimicrobial resistance profiles and resistance genes in *Vibrio parahaemolyticus* isolates from Zhanjiang shrimp aquaculture farms and evaluated their association with bacteriophage susceptibility. A total of 132 isolates were tested against 18 antibiotics, while molecular and phage-related analyses were performed on 132 isolates. High resistance was observed against kanamycin (95.5%), sulfamethoxazole (94.7%), cefazolin (78.8%), imipenem (78.8%), amoxicillin (70.5%), and ampicillin (65.2%). Multidrug resistance was detected in 131/132 isolates (99.24%), indicating an extensive resistance burden. PCR analysis showed high detection rates of *aphA* (100.00%), *sul1* (99.24%), *blaTEM* (98.48%), *blaOXA* (73.48%), *blaCTX-M* (53.03%), *qnrA* (45.45%), *tetA* (37.88%), and *tetB* (21.21%). Phage response analysis showed that 70.45% and 68.18% of isolates were phage-resistant at 4 h and 5 h, respectively. Genes potentially associated with phage–host interactions were also detected, with *luxS* showing the highest frequency (85.61%). Association analysis revealed significant links between *tetA*, *tetB*, *blaOXA*, and bacteriophage responses. These findings suggest that antimicrobial resistance and reduced phage susceptibility can co-occur among *V. parahaemolyticus* isolates recovered from shrimp farms in Zhanjiang, highlighting the need for regional resistance surveillance and careful phage selection.

## 1. Introduction

*Vibrio parahaemolyticus* is a halophilic Gram-negative bacterium that is usually found in marine and estuarine environments and is widely detected in fish, shellfish, and shrimp products worldwide [1,2]. Because consumption of raw or undercooked seafood contaminated with pathogenic *V. parahaemolyticus* can cause acute gastroenteritis and even death in severe cases [3], it is considered as important foodborne pathogen. In addition to food safety, *V. parahaemolyticus* is also a major concern in aquaculture, where it occurs naturally in aquatic systems and acts as an opportunistic pathogen in shrimp and other aquatic animals, leading to disease outbreaks, mass mortality and serious losses in production [4,5].

Aquaculture is one of the fastest-growing food-producing sectors globally, but intensive production systems have increased the risk of bacterial disease outbreaks, particularly vibriosis [6]. Among *Vibrio* species, *V. parahaemolyticus*, *V. vulnificus*, and *V. harveyi* are frequently associated with severe diseases in marine and brackish aquaculture systems and may also pose risks to human health through the seafood chain [7]. Therefore, the occurrence of *V. parahaemolyticus* in shrimp aquaculture should be considered not only an animal health problem but also a one-health issue linking aquaculture productivity, seafood safety, and public health [8].

Antibiotics are widely used to control bacterial infections in aquaculture; however, excessive or inappropriate use has contributed to the emergence and evolution of antibiotic-resistant *V. parahaemolyticus* [9,10]. Previous studies have reported that *V. parahaemolyticus* isolates are commonly resistant to antibiotics such as ampicillin, kanamycin, and streptomycin, and multidrug-resistant isolates have been recovered from seafood and aquaculture environments [11,12]. The presence of multidrug-resistant pathogens in aquaculture environments is particularly concerning because they can enter aquatic organisms through the food chain and subsequently pose risks to both aquaculture and human health [13,14].

The genetic basis of antimicrobial resistance in *V. parahaemolyticus* is often linked to antibiotic resistance genes located on chromosomes, plasmids, and other mobile genetic elements [2,15]. Mobile genetic elements facilitate the accumulation and spread of resistance genes through horizontal gene transfer, whereas conjugative plasmids are important vectors for the dissemination of antibiotic resistance genes [2,16]. For example, Wang et al. [15] reported a multidrug-resistant *V. parahaemolyticus* strain carrying resistance genes, including *tetB*, *tetM*, *sul2*, *qnrVC6*, *floR*, and *blaCARB*, with several genes surrounded by transposase genes, suggesting that plasmids and transposons may contribute to the dissemination of resistance genes [17]. Similarly, Sony et al. [7] identified *tetB* and *tetH* as predictors of tetracycline resistance in fish-pathogenic Vibrio isolates, supporting the need to connect phenotypic resistance patterns with detected resistance genes.

Due to increasing concerns regarding antibiotic resistance, bacteriophage therapy has been proposed as an alternative or complementary strategy for controlling pathogenic bacteria in aquaculture [18,19]. Phage therapy uses bacteriophages to kill specific bacteria through their natural viral life cycle. Phages are attractive because they usually show high specificity toward bacterial targets and multiply during infection [8,20]. In aquaculture and seafood depuration systems, phages may help reduce *Vibrio* loads while limiting the disruption of the wider microbiota. Recent studies on *V. parahaemolyticus* phages have shown that isolated phages can reduce bacterial counts by more than 4 log CFU/mL under suitable conditions, demonstrating their potential for bacterial control [8].

However, the emergence of phage-resistant bacteria remains an important bottleneck for phage-based control strategies [21]. Phage resistance may arise through changes in bacterial receptors, surface structures, defense systems, or other bacterial mechanisms that prevent phage adsorption or replication [22]. Importantly, phage resistance can also carry fitness costs, including reduced growth, altered motility, changes in antibiotic resistance, or reduced virulence [23,24]. Duarte et al. [8] reported that phage-resistant *V. parahaemolyticus* mutants emerged at low frequency, resistance was not always permanent, and resistant mutants showed reduced bacterial fitness, indicating that the biological cost of phage resistance should be considered when evaluating phage therapy.

Although previous studies have separately examined antimicrobial resistance or bacteriophage control in *V. parahaemolyticus*, fewer studies have evaluated phenotypic antibiotic resistance, multidrug resistance, antibiotic resistance genes, bacteriophage response, and genes potentially associated with phage–host interactions within the same isolate collection. This gap is important for shrimp aquaculture, where both antibiotic exposure and phage-based control strategies may impact bacterial survival and adaptation. Therefore, the current study was conducted to determine the antimicrobial susceptibility profiles, multidrug resistance patterns, distribution of antibiotic resistance genes, bacteriophage susceptibility, and genes potentially associated with phage–host interactions in *V. parahaemolyticus* isolates. The study also assessed the correlation between the identified resistance genes and phenotypic resistance and between the antibiotic resistance genes and the bacteriophage response. Therefore, this study aimed to characterize antimicrobial resistance profiles and resistance genes among *V. parahaemolyticus* isolates recovered from shrimp aquaculture farms in Zhanjiang, China, and to investigate the association between detected resistance determinants and susceptibility responses to an isolated bacteriophage.

## 2. Materials and Methods

### 2.1. Sample Collection

**Figure 1:**
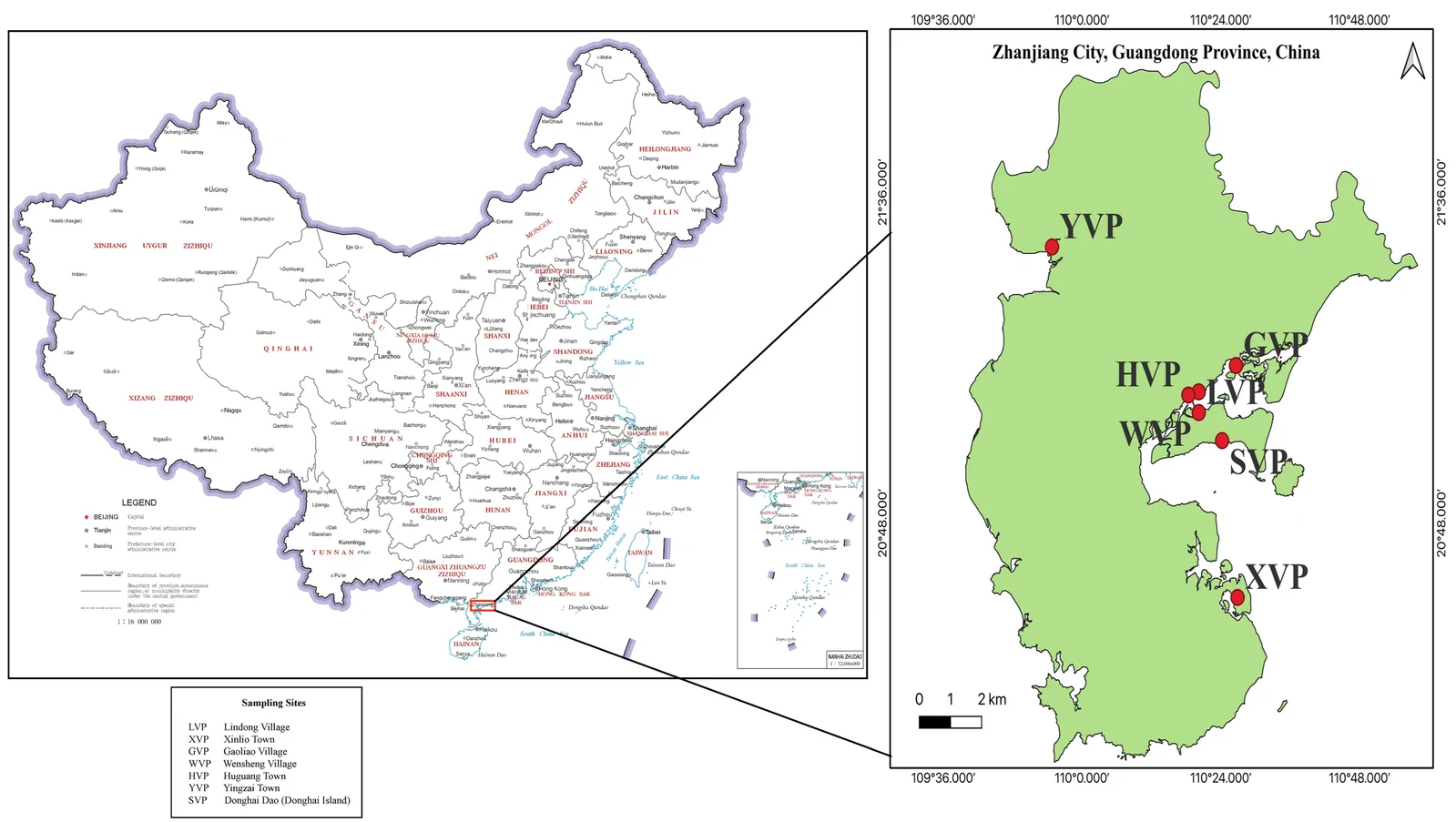
Spatial distribution of sampling locations across seven aquatic sites in Zhanjiang, China. Map modified from (Standard Map Service of China).

Samples of shrimp intestine, pond sediment and aquaculture water were collected from seven aquaculture farm sites in Zhanjiang, China, namely Huguang Town, Lindong Village, Wensheng Village, Xinliao Town, Yingzai Town, Gaoliao Village, and Donghai Dao (Figure 1; Table 1). Each sample was collected separately in sterile plastic bags to prevent cross-contamination. All samples were cooled immediately after collection and transported to the microbiology laboratory at 7–10 °C for further analysis.

### 2.2. Isolation and Identification of Vibrio parahaemolyticus

A total of 89 samples were collected from shrimp aquaculture environments, including 14 shrimp intestine samples, 33 sediment samples, and 42 aquaculture water samples. Following selective isolation, three presumptive *Vibrio* isolates were obtained from each sample, resulting in 267 recovered isolates. After phenotypic screening, MALDI-TOF MS identification, and molecular confirmation by 16S rRNA gene sequencing, 132 isolates were identified as *Vibrio parahaemolyticus*, including 60 isolates from sediment samples, 56 from water samples, and 16 from shrimp intestine samples (Table 2). Isolation was conducted following the Chinese National Food Safety Standard GB 4789.7-2013 with minor modifications (GB 4789.7-2013) [25]. Similar selective isolation procedures have been applied for the recovery of *Vibrio* species from aquaculture and seafood samples [2,7].

First, 10 g of sediment was suspended in sterile distilled water and 20 g of shrimp intestine tissue was aseptically homogenized in sterile phosphate-buffered saline. Water sample processing was done on the premises. Shrimp supernatant, sediment suspension and pond water were streaked on thiosulfate citrate bile salts sucrose agar and incubated for 18 h at 37 °C. Presumed *V. parahaemolyticus* colonies were picked based on typical green to blue-green colony morphology on TCBS agar.

The colonies were grown overnight at 37 °C in Luria–Bertani broth with shaking. Isolates were stored in pure culture at −80 °C in LB broth supplemented with glycerol. Initial characterization was performed based on colony morphology, halophilic growth characteristics, and biochemical properties. Species-level identification was subsequently conducted using matrix-assisted laser desorption/ionization time-of-flight mass spectrometry (MALDI-TOF MS, Bruker Daltonics, Bremen, Germany). The obtained mass spectra were compared with the reference database, and isolates showing reliable spectral matches consistent with *V. parahaemolyticus* were selected for subsequent analyses.

To further support species identification, genomic DNA was extracted from MALDI-TOF MS-identified isolates, and amplification and sequencing of the 16S rRNA gene were performed using universal primers 27F and 1492R [26]. The obtained sequences were compared with reference sequences available in the NCBI nucleotide database using BLASTn (Basic Local Alignment Search Tool; https://blast.ncbi.nlm.nih.gov/Blast.cgi, accessed on 22 December 2024). The final identification of *V. parahaemolyticus* isolates was determined based on the combined evidence from MALDI-TOF MS identification, 16S rRNA gene sequence similarity, and consistent phenotypic characteristics [26,27].

### 2.3. Antibiotic Susceptibility Testing

In accordance with the Clinical and Laboratory Standards Institute recommendations, the broth microdilution minimum inhibitory concentration method was used to assess the antimicrobial susceptibility of 132 confirmed *V. parahaemolyticus* isolates [28]. Antimicrobial resistance in *V. parahaemolyticus* from aquatic animal and seafood sources has been extensively assessed using MIC-based techniques [2].

Before testing, frozen bacterial stocks were revived to normal conditions in Mueller–Hinton broth supplemented with 2% NaCl and cultured for 18–24 h at 37 °C. Eighteen antibiotics were tested: tetracycline, doxycycline, ciprofloxacin, ofloxacin, levofloxacin, chloramphenicol, ampicillin, amoxicillin, ceftazidime, cefazolin, cefuroxime sodium salt, cefotaxime, cefoxitin, imipenem, meropenem, gentamicin, sulfamethoxazole, and kanamycin.

The initial stock concentrations and antibiotic-specific two-fold serial dilution ranges used for MIC determination are provided in Supplementary Table S1. Each antibiotic was prepared individually according to the manufacturer’s recommendations and tested within its corresponding dilution range to ensure accurate interpretation according to antibiotic-specific susceptibility criteria.

Antibiotic stock solutions were prepared at an initial concentration of 5120 μg/mL, sterilized using 0.22 µm membrane filters where required, and stored at −20 °C until use. Working antibiotic solutions were prepared by two-fold serial dilution in Mueller–Hinton broth supplemented with 2% NaCl, with the final testing concentrations adjusted according to the specific dilution range of each antimicrobial agent (Supplementary Table S1).

Bacterial suspensions were adjusted to the 0.5 McFarland standard, yielding a final inoculum of approximately 5 × 10^5^ CFU/mL per well. Antibiotic solutions and bacterial suspensions were transferred into sterile 96-well microtiter plates for MIC determination. Antibiotic-free growth controls containing bacterial suspension without antibiotics and bacteria-free sterility controls containing only culture medium were included in each assay. Plates were incubated for 18–24 h at 37 °C.

The MIC is the lowest antibiotic concentration at which no discernible bacterial growth occurs. The MIC values were interpreted according to the Clinical and Laboratory Standards Institute (CLSI) guideline M45, 3rd Edition, for *Vibrio* spp. where applicable. Isolates were categorized as susceptible, intermediate, or resistant based on the specific MIC interpretive criteria provided for each antimicrobial agent. The breakpoint sources and corresponding S/I/R criteria used for interpretation are summarized in Supplementary Table S2. For antimicrobial agents without specific *Vibrio* spp. interpretive criteria, available CLSI recommendations or relevant published criteria were applied, as indicated in Supplementary Table S2A. For antimicrobial agents without specific *Vibrio* spp. interpretive criteria in CLSI M45, available CLSI M100 criteria were applied [28]. Multidrug resistance (MDR) was defined as resistance to at least one antimicrobial agent in three or more antimicrobial classes, according to the internationally accepted definition proposed by Magiorakos et al. and Xiang et al. [29,30]. MDR classification was determined based on the S/I/R interpretation obtained from the MIC breakpoint criteria listed in Supplementary Table S2A. *Escherichia coli* ATCC 25922 was used as the quality control strain. All assays were performed in triplicate.

### 2.4. Phage Isolation, Propagation and Titer Determination

Phage-containing samples with potential lytic activity against *V. parahaemolyticus* were obtained from aquaculture water and sediment samples using an enrichment method. Phage enrichment and double-layer agar plaque assays were performed following previously described bacteriophage isolation procedures, with slight modifications [8,31].

Briefly, 10 mL of aquaculture water or 10 g of sediment suspension in sterile saline was centrifuged at 8000× *g* for 10 min to remove particulate matter. The supernatant was mixed with log-phase-sensitive *V. parahaemolyticus* host strains in alkaline peptone water containing 3.5% NaCl. The mixture was incubated at 37 °C for 18–24 h with shaking at 120–150 rpm to allow phage adsorption and replication. The enriched cultures were then centrifuged and filtered through 0.22 µm membrane filters to obtain cell-free phage lysates. The lysates were stored at 4 °C for short-term use.

Phage titers were determined using the double-layer agar plaque assay. Phage lysates were serially diluted ten-fold in sterile saline. Aliquots of each dilution were mixed with fresh host bacterial culture and molten soft agar, and then poured onto tryptic soy agar plates supplemented with 3.5% NaCl. Plates were incubated at 37 °C for 16–18 h. Plaques were counted, and phage concentrations were expressed as plaque-forming units per milliliter. The same environmental water and sediment samples collected from each aquaculture sampling location were used for both *V. parahaemolyticus* isolation and bacteriophage enrichment. Therefore, each recovered bacteriophage preparation originated from the same environmental niche as the corresponding bacterial isolates. The *V. parahaemolyticus* isolates obtained from each sampling location were used as host strains for phage propagation and subsequent susceptibility evaluation. A total of seven bacteriophage isolates were recovered from the seven sampling locations. Each bacteriophage preparation was evaluated against the collection of *V. parahaemolyticus* isolates obtained from its corresponding sampling location to characterize site-specific phage–host susceptibility patterns.

The susceptibility response of each bacterial isolate was assessed based on phage-mediated growth inhibition using preliminary spot screening followed by quantitative analysis using a 96-well microplate assay (Thermo Fisher Scientific, Waltham, MA, USA).

### 2.5. Preliminary Phage Susceptibility Screening by Spot Assay

The spot assay was performed as a preliminary qualitative screening method to identify potential phage-sensitive *V. parahaemolyticus* isolates. Because spot lysis may represent either productive phage infection or non-productive lysis from high phage concentrations, spot assay results were not used as the final criterion for susceptibility classification. The spot assay method, which is widely used for preliminary screening of phage susceptibility among bacterial isolates, was performed to evaluate the susceptibility spectrum of isolated bacteriophages against 132 confirmed *Vibrio parahaemolyticus* isolates (Supplementary Figure S1) [32,33]. In this study, seven bacteriophage isolates were used for susceptibility screening. These phages were isolated from the same aquaculture sampling locations where the environmental *V. parahaemolyticus* isolates were collected, allowing evaluation of phage–host interactions under site-specific ecological conditions. Each phage preparation was tested individually against the bacterial collection to determine phage-specific inhibitory activity and characterize the susceptibility patterns of environmental *V. parahaemolyticus* isolates. The spot assay was used as an initial qualitative screening approach to identify potentially susceptible bacterial isolates before quantitative confirmation using the 96-well microplate growth inhibition assay.

Each *V. parahaemolyticus* isolate was cultured at 37 °C with shaking in tryptic soy broth containing 3.5% NaCl until reaching the exponential growth phase. The bacterial suspension was adjusted to approximately 0.5 McFarland standard. Tryptic soy agar supplemented with 3.5% NaCl was overlaid with 100 µL of standardized bacterial culture mixed with 3 mL of molten soft agar.

Ten microliters of phage lysate (approximately 10^6^–10^8^ PFU/mL) were spotted onto each bacterial lawn. Plates were air-dried under aseptic conditions and incubated at 37 °C for 18–24 h. Clear and well-defined lysis zones were considered preliminary evidence of phage activity against the tested isolates. However, because spot assays may detect both productive phage infection and non-productive lysis caused by high phage concentrations, spot assay results were used only for preliminary screening. Isolates showing potential susceptibility were further evaluated using a quantitative 96-well microplate growth inhibition assay, and final susceptibility classification was based on OD600 reduction compared with untreated controls.

Turbid or incomplete lysis indicated reduced susceptibility, whereas absence of visible lysis was recorded as no detectable susceptibility under the tested conditions. All assays were performed in triplicate.

Isolates showing visible lysis zones in the spot assay were subsequently subjected to quantitative growth inhibition analysis using the 96-well microplate assay (Supplementary Table S4).

### 2.6. Quantitative Assessment of Phage-Mediated Growth Inhibition Using a 96-Well Microplate Assay

The quantitative 96-well microplate growth inhibition assay was used as the final method for evaluating phage susceptibility. The assay was selected because it provides a quantitative measurement of bacterial growth suppression following phage exposure and allows comparison between phage-treated and untreated cultures.

The inhibitory effects of phage lysates against confirmed *Vibrio parahaemolyticus* isolates were quantitatively evaluated using a 96-well microplate growth inhibition assay. This approach was applied to determine bacterial susceptibility by monitoring changes in bacterial growth after phage exposure. Briefly, overnight cultures of *V. parahaemolyticus* isolates were grown in tryptic soy broth supplemented with 3.5% NaCl and adjusted to approximately 10^6^ CFU/mL before the assay. A standardized volume of bacterial suspension was mixed with phage lysates at selected multiplicity of infection (MOI) values in sterile 96-well microplates. The MOI was calculated as the ratio of phage particles (PFU/mL) to bacterial cells (CFU/mL). A standardized MOI of 0.1 was used for all phage susceptibility experiments. Briefly, bacterial cultures were adjusted to approximately CFU/mL and mixed with phage lysates containing approximately PFU/mL.

Wells containing bacterial cultures without phage addition were used as untreated growth controls, while medium-only wells served as blank controls. The plates were incubated at 37 °C, and bacterial growth was monitored by measuring optical density at 600 nm (OD600) at regular intervals using a microplate reader (Supplementary Tables S3 and S4).

The reduction in OD600 values compared with untreated controls was calculated to determine phage-mediated growth inhibition. The degree of phage-mediated growth inhibition was calculated based on the reduction in OD600 values compared with untreated bacterial controls. Growth inhibition (%) was calculated according to the following equation:

$$Growth\;inhibition\;(\%)=\frac{ODcontrol-ODphage-treated}{ODcontrol}\;\times 100$$

Isolates showing a reproducible reduction in bacterial growth compared with untreated controls were considered susceptible to the tested phage preparation, whereas isolates showing no measurable inhibition were considered non-susceptible under the evaluated conditions. Because phage activity can vary depending on phage–host combinations, susceptibility classification was restricted to the specific phage preparations tested in this study.

This approach has been widely applied for quantitative evaluation of bacteriophage-mediated bacterial growth inhibition in microplate assays [34,35].

All experiments were performed in biological triplicates, and growth inhibition was evaluated based on reproducible differences between phage-treated and untreated bacterial cultures. Susceptibility classification was based on the quantitative reduction in bacterial growth determined from OD600 measurements rather than spot assay observations alone.

The relative growth ratio was calculated as the OD600 value of phage-treated cultures divided by the OD600 value of untreated bacterial controls. A ratio ≥ 1 indicated that phage exposure did not reduce bacterial growth compared with the untreated control, whereas a ratio < 1 indicated measurable growth inhibition by the tested phage preparation. The resulting growth inhibition profiles were used for subsequent classification of phage response patterns. This threshold was applied as an operational criterion to distinguish isolates showing detectable phage-mediated growth inhibition from those showing no measurable inhibition under the tested experimental conditions. Because phage susceptibility represents a continuous response, isolates with values close to the threshold were interpreted cautiously.

### 2.7. Selection of Isolates Exhibiting Reduced Phage Susceptibility

Isolates exhibiting reduced susceptibility to phage exposure were identified based on the quantitative 96-well microplate growth inhibition assay. The spot assay results were used only as an initial qualitative screening step to identify potential phage activity and guide subsequent quantitative evaluation. Each isolate was evaluated using three independent replicate measurements. For each replicate, the relative growth ratio was calculated as the OD600 value of the phage-treated culture divided by the OD600 value of the untreated control. A replicate was classified as exhibiting reduced susceptibility when the OD600 ratio was ≥1 and as susceptible when the OD600 ratio was <1. The final isolate-level susceptibility phenotype was determined using a majority-rule approach, where isolates showing reduced susceptibility in at least two of the three replicates were classified as exhibiting reduced susceptibility. Isolates that showed no lysis or turbid lysis after exposure to the tested phages were considered to exhibit reduced susceptibility or partial susceptibility and were selected for further molecular screening. The interpretation of phage resistance was based on previously described methods for evaluating bacterial susceptibility and resistance to phage infection [8].

Phage resistance can occur through changes in bacterial surface receptors, lipopolysaccharide structure, restriction–modification systems, quorum sensing regulation, or other bacterial defense mechanisms [22]. Therefore, the selected isolates were further analyzed for the presence of phage resistance-associated genes.

### 2.8. PCR Detection of Antibiotic Resistance Genes

Genomic DNA was extracted from overnight bacterial cultures using a Rapid Bacterial Genomic DNA Isolation Kit (Solarbio Life Sciences, Beijing, China) according to the manufacturer’s instructions. PCR was used to detect selected antibiotic resistance genes among the confirmed *V. parahaemolyticus* isolates. The screened genes included *aphA*, *blaCTX-M*, *blaOXA*, *blaTEM*, *qnrA*, *sul1*, and *tetA* and *tetB*. These genes were selected because they are associated with resistance to aminoglycosides, β-lactams, quinolones, sulfonamides, and tetracyclines, which are commonly reported in *Vibrio* species and aquaculture-associated bacteria (Supplementary Dataset S1) [36,37].

PCR reactions were performed in a final volume of 25 µL containing 12.5 µL of 2× SanTaq PCR Mix, 1 µL of forward primer, 1 µL of reverse primer, 9.5 µL of sterile distilled water, and 1 µL of DNA template. The primer sequences, target genes, and expected amplicon sizes used for detection of antimicrobial resistance genes (*tetA*, *tetB*, *blaTEM*, *blaCTX-M*, *blaOXA*, *qnrA*, *sul1*, and *aphA*) are listed in Supplementary Table S5. The cycling conditions included initial denaturation at 94 °C for 5 min, followed by 35 cycles of denaturation at 94 °C for 30 s, annealing at the specific temperature for each primer set, extension at 72 °C for 30 s and final extension at 72 °C for 10 min. A negative control containing sterile distilled water instead of DNA template was included in each PCR run to monitor potential contamination and ensure the reliability of amplification results. The annealing temperatures were 56.4 °C for *tetA* and *tetB*, *blaTEM*, and *blaCTX-M*; 55.7 °C for *sul1*, *aphA*, and *blaOXA*; and 53.5 °C for *qnrA*. PCR products were analyzed by electrophoresis on 2.0% agarose gels in 1× TAE buffer at 124 V and 94 mA for approximately 20 min. A 5000 bp DNA ladder was used as the molecular size marker. Bands of the expected size were visualized using a gel imaging system and recorded as positive amplification.

### 2.9. PCR Detection of Genes Potentially Associated with Bacteriophage Susceptibility and Resistance

Four bacterial genes potentially involved in bacteriophage susceptibility and resistance mechanisms, including *hsdM*, *luxS*, *rfb*, and *ugd*, were selected for PCR detection. These genes were not considered phage-specific resistance determinants but were selected based on their reported roles in bacterial processes that may influence phage infection outcomes. The *hsdM* gene is associated with restriction–modification systems that can limit foreign DNA replication, including phage genomes. The *rfb* and *ugd* genes participate in lipopolysaccharide (LPS) biosynthesis and modification, which can influence phage adsorption by altering bacterial surface receptors. The *luxS* gene was included because AI-2 quorum sensing mediated by *LuxS* can regulate biofilm formation, bacterial stress responses, and physiological states that may indirectly affect phage susceptibility [38,39,40].

The same reaction combination used for antibiotic resistance genes was used for PCR amplification. Initial denaturation at 94 °C for 5 min, subsequent 35 cycles of denaturation at 94 °C for 30 s, annealing at 56.2 °C for *luxS* and *hsdM* or 54.5 °C for *rfb* and *ugd*, extension at 72 °C for 30 s, and final extension at 72 °C for 10 min were the cycling conditions. Phage–host interaction-associated genes (*luxS*, *rfb*, *hsdM*, and *ugd*) are listed in Supplementary Table S4. Gel imaging equipment was used to visualize the separated PCR results on 2.0% agarose gels. Positive bands were those that matched the anticipated amplicon sizes.

### 2.10. Data Analysis

The detection level of Vibrio parahaemolyticus among different sample types was analyzed using the chi-square test in SPSS 20.0 statistical software (SPSS Inc., Chicago, IL, USA). Spearman correlation analysis was used to evaluate the relationship between antimicrobial resistance phenotypes and the corresponding antibiotic resistance genes.

The association between antibiotic resistance genes and phage response was analyzed using Fisher’s exact test. Since phage response was evaluated at two different time points (4 h and 5 h) using the same bacterial isolates, the associations were assessed separately for each time point. To reduce the potential influence of multiple comparisons, *p*-values were adjusted using the Benjamini–Hochberg false discovery rate correction. Adjusted *p*-values (q-values) < 0.05 were considered statistically significant.

## 3. Results

### 3.1. Antibiotic Resistance Profiles of Vibrio parahaemolyticus Isolates

**Figure 2:**
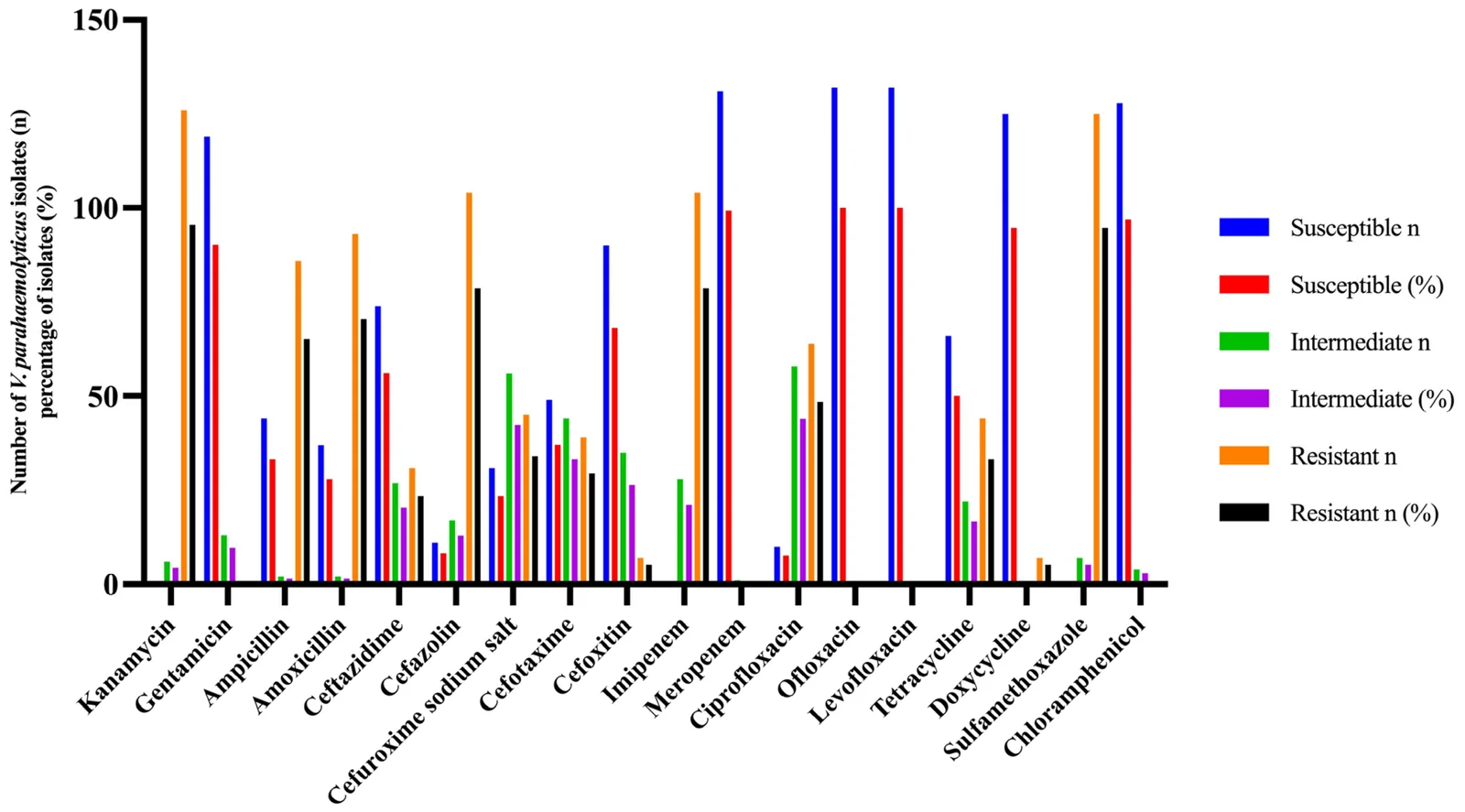
Antimicrobial susceptibility profile of *Vibrio parahaemolyticus* isolates. The *y*-axis represents the number of *V. parahaemolyticus* strains (n) and the corresponding percentage distribution (%), while different colors indicate susceptible (S), intermediate (I), and resistant (R) responses. Here, S(n), I(n), and R(n) represent the number of susceptible, intermediate, and resistant isolates, respectively, whereas S(%), I(%), and R(%) indicate the corresponding percentages of isolates. The susceptibility testing results showed marked variation in the response of the 132 *V. parahaemolyticus* isolates to the 18 antimicrobials tested. All isolates were sensitive to ofloxacin and levofloxacin, with high susceptibility to meropenem (99.2%), chloramphenicol (97.0%), doxycycline (94.7%), and gentamicin (90.2%). Cefoxitin, ceftazidime, tetracycline, and cefotaxime all showed moderate susceptibility, indicating varying antibiotic effectiveness. In contrast, kanamycin (95.5%), sulfamethoxazole (94.7%), cefazolin (78.8%), imipenem (78.8%), amoxicillin (70.5%), and ampicillin (65.2%) all had significant resistance rates. Ciprofloxacin and cefuroxime sodium salt demonstrated reduced efficacy, with poor susceptibility and significant intermediate or resistance responses (Supplementary Table S2). The study found that fluoroquinolones and antibiotics like meropenem, chloramphenicol, doxycycline, and gentamicin are highly effective against the isolates. However, resistance to sulfonamides, β-lactams, and some aminoglycosides suggests the presence of multidrug-resistant *V. parahaemolyticus*, which could impact food safety and public health.

Eighteen antibiotics were evaluated against 132 *V. parahaemolyticus* isolates recovered from shrimp farms in Zhanjiang, China. The isolates displayed inconsistent patterns of susceptibility to all the antibiotics that were examined (Table 3; Figure 2). These findings indicate that the isolates showed high resistance to several commonly used antibiotics, particularly sulfonamides, aminoglycosides and β-lactams, while some fluoroquinolones and other antibiotics remained highly active.

#### Recovery and Identification of *Vibrio parahaemolyticus* Isolates

A total of 132 confirmed *V. parahaemolyticus* isolates were recovered from 89 aquaculture samples. Sediment samples yielded the highest number of confirmed isolates (60/99, 60.6%), followed by water samples (56/126, 44.4%) and shrimp intestine samples (16/42, 38.1%). The distribution of recovered isolates among different sampling sites and sample types is summarized in Table 2.

### 3.2. Multidrug Resistance and MAR Index

Multidrug resistance was prevalent among *V. parahaemolyticus* isolates recovered from Zhanjiang shrimp aquaculture farms. According to the MDR criteria proposed by Magiorakos et al. and Xiang et al. [29,30], isolates were classified as multidrug-resistant when they exhibited resistance to at least one antimicrobial agent belonging to three or more antimicrobial categories. Based on this classification, 131 of the 132 isolates (99.24%) were identified as MDR, whereas only one isolate (0.76%) did not meet the MDR criterion. The number of resistant antimicrobial categories among individual isolates ranged from one to seven, while the MAR index values ranged from 0.06 to 0.56.

**Figure 3:**
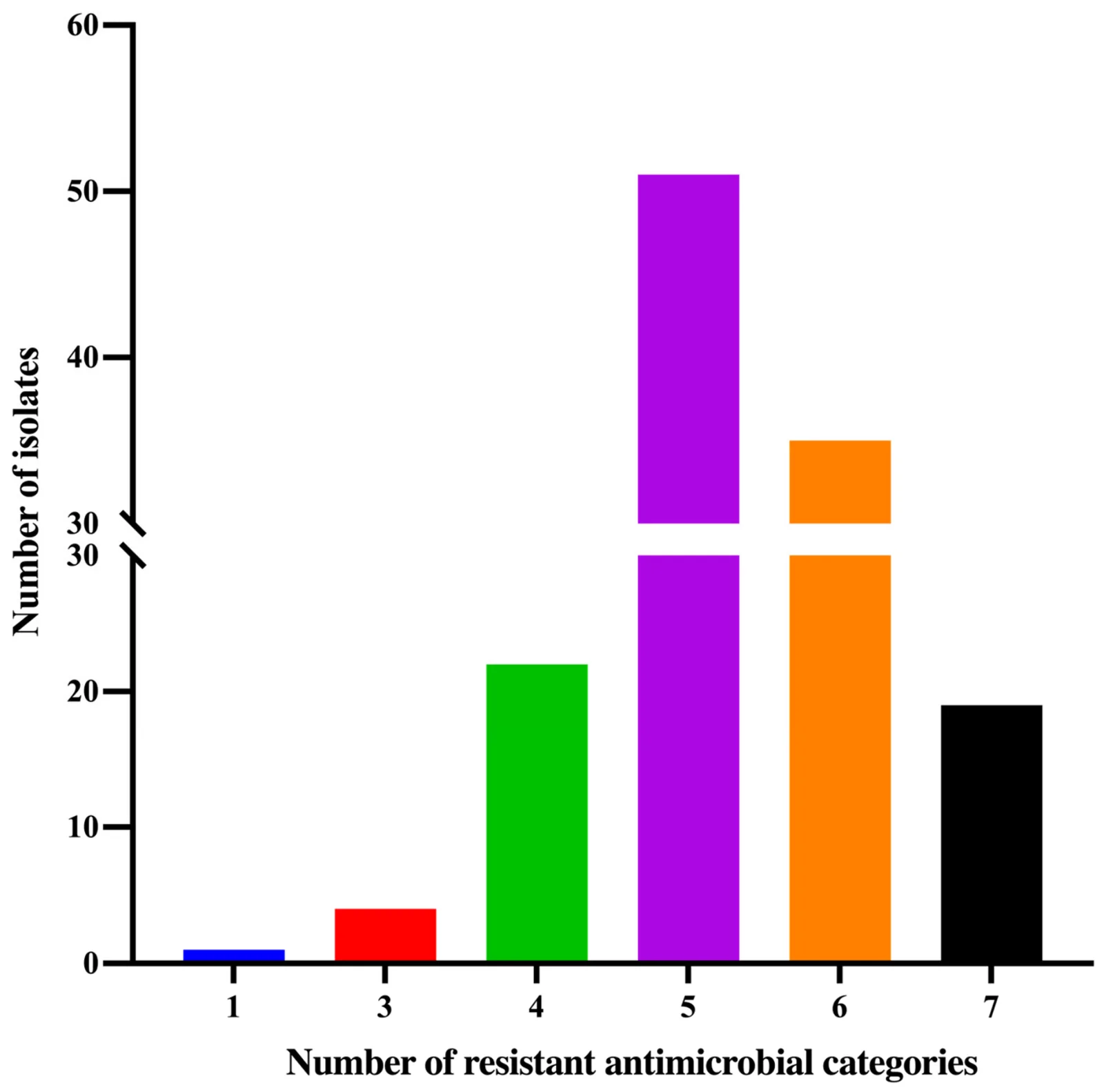
Distribution of multidrug resistance patterns based on antimicrobial categories among 132 *Vibrio parahaemolyticus* isolates. The *x*-axis represents the number of antimicrobial categories to which each isolate exhibited resistance, while the *y*-axis indicates the number of isolates showing resistance at each category level. Multidrug resistance (MDR) was defined as acquired resistance to at least one antimicrobial agent in three or more antimicrobial categories according to the criteria proposed by Magiorakos et al. [29]. Among the 132 tested isolates, 131 (99.24%) were classified as MDR. Resistance to five antimicrobial categories was the most frequently observed pattern, detected in 51 isolates (38.64%), followed by resistance to six categories in 35 isolates (26.52%) and four categories in 22 isolates (16.67%). Nineteen isolates (14.39%) showed resistance to seven antimicrobial categories, whereas four isolates (3.03%) exhibited resistance to three antimicrobial categories. Only one isolate (0.76%) did not meet the MDR criterion.

Among the MDR isolates, resistance across five antimicrobial categories was the most frequently observed pattern, occurring in 51 isolates (38.64%), followed by resistance to six categories in 35 isolates (26.52%) and four categories in 22 isolates (16.67%). Nineteen isolates (14.39%) exhibited resistance to seven antimicrobial categories, representing the highest resistance category observed in this study. In contrast, four isolates (3.03%) showed resistance to three antimicrobial categories (Figure 3). Overall, these findings demonstrate that *V. parahaemolyticus* isolates recovered from Zhanjiang shrimp aquaculture farms exhibited extensive multidrug resistance across multiple antimicrobial categories, highlighting potential risks associated with antimicrobial dissemination in aquaculture environments and seafood production systems.

### 3.3. Phage Susceptibility and Resistance Profiles

The phage susceptibility of 132 *Vibrio parahaemolyticus* isolates was analyzed. The response of bacterial isolates to phage exposure was estimated by measuring the changes in bacterial growth (OD_600_) at 4 and 5 h post phage treatment. Optical density measurements in growth inhibition assays are widely used to assess bacteriophage activity and to classify bacterial susceptibility responses to phages [41,42]. All experiments were carried out in triplicate, and the final classification of each isolate was based on the predominant response from the replicate measurements at each time point.

After exposure to phage, responses to growth allowed for the separation of isolates into phage-susceptible and phage-resistant groups. Phage-resistant isolates were defined as those that continued to grow in the presence of phage; thus, the phage tested could not effectively inhibit and lyse the bacterial cells. In contrast, phage-susceptible isolates were described as those with apparent growth inhibition post phage treatment, which indicated efficient phage-mediated bacterial control [43,44].

**Figure 4:**
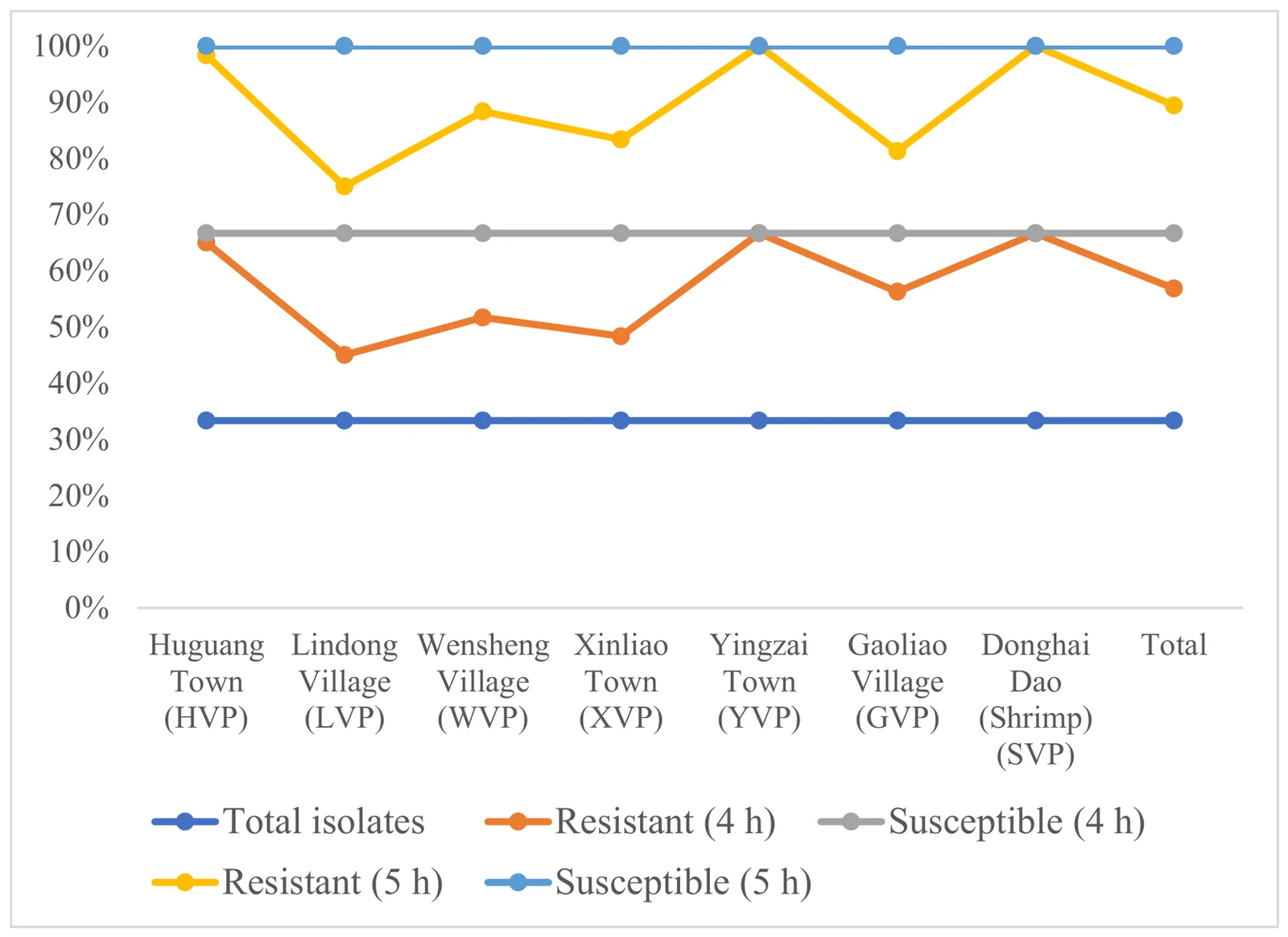
Sampling-site-specific prevalence of phage-resistant and phage-susceptible *Vibrio parahaemolyticus* isolates at 4 h and 5 h. Isolate phenotypes were assigned based on the majority response of three replicates, with OD_2_/OD_1_ ≥ 1 classified as resistant and OD_2_/OD_1_ < 1 classified as susceptible. Values represent the percentage of isolates exhibiting resistance or susceptibility at each sampling site [35,45].

Ninety-three isolates (70.45%) were defined as phage-resistant, and 39 isolates (29.55%) were sensitive to phage treatment at 4 h post phage exposure. At 5 h, 90 isolates (68.18%) were still resistant, and 42 isolates (31.82%) were susceptible (Table 4; Figure 4). These findings indicate that a substantial proportion of *V. parahaemolyticus* isolates exhibited reduced susceptibility to the tested bacteriophage, suggesting potential challenges for phage-based biocontrol applications in aquaculture systems. Detailed replicate-level phage response data are provided in Supplementary Tables S6 and S7.

### 3.4. Detection of Antibiotic Resistance Genes

**Figure 5:**
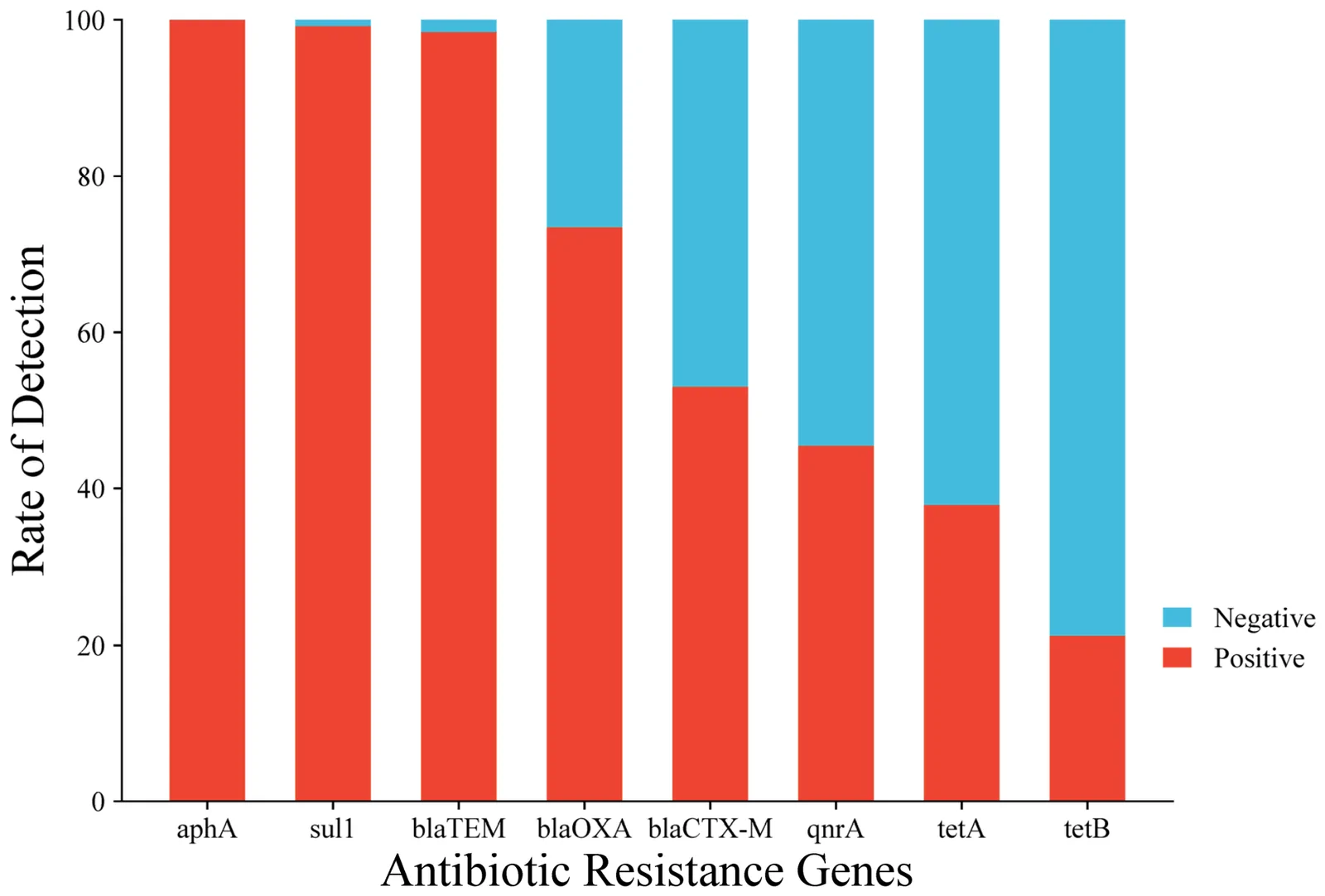
Detection rates of antibiotic resistance genes in *Vibrio parahaemolyticus* isolates. The figure shows the distribution of selected antibiotic resistance genes among 132 *V. parahaemolyticus* isolates. The highest detection rate was recorded for *aphA* (100%), followed by *sul1* (99.24%) and *blaTEM* (98.48%), indicating widespread aminoglycoside, sulfonamide, and β-lactam resistance gene carriage. The β-lactam resistance gene *blaOXA* was also frequently detected (73.48%), as well as *blaCTX-M* (53.03%). In contrast, *qnrA*, associated with fluoroquinolone resistance, was detected in 45.45% of isolates, suggesting a moderate occurrence of plasmid-mediated quinolone resistance. The tetracycline resistance genes *tetA* (34.09%) and *tetB* (21.21%) showed lower detection rates. Overall, the findings indicate a high prevalence of resistance genes among *V. parahaemolyticus* isolates, particularly those associated with aminoglycoside, sulfonamide, and β-lactam resistance.

PCR detection of antibiotic resistance genes was performed on 132 isolates that produced interpretable amplification results. The most frequently detected gene was *aphA*, which was present in all 132 isolates (100%). The *sul1* gene was detected in 131 isolates (99.24%), while *blaTEM* was detected in 130 isolates (98.48%). Other detected genes included *blaOXA* in 97 isolates (73.48%), *blaCTX-M* in 70 isolates (53.03%), *qnrA* in 60 isolates (45.45%), *tetA* in 50 isolates (37.88%) and *tetB* in 28 isolates (21.21%) (Table 5; Figure 5).

### 3.5. Detection of Genes Potentially Associated with Phage Susceptibility

**Figure 6:**
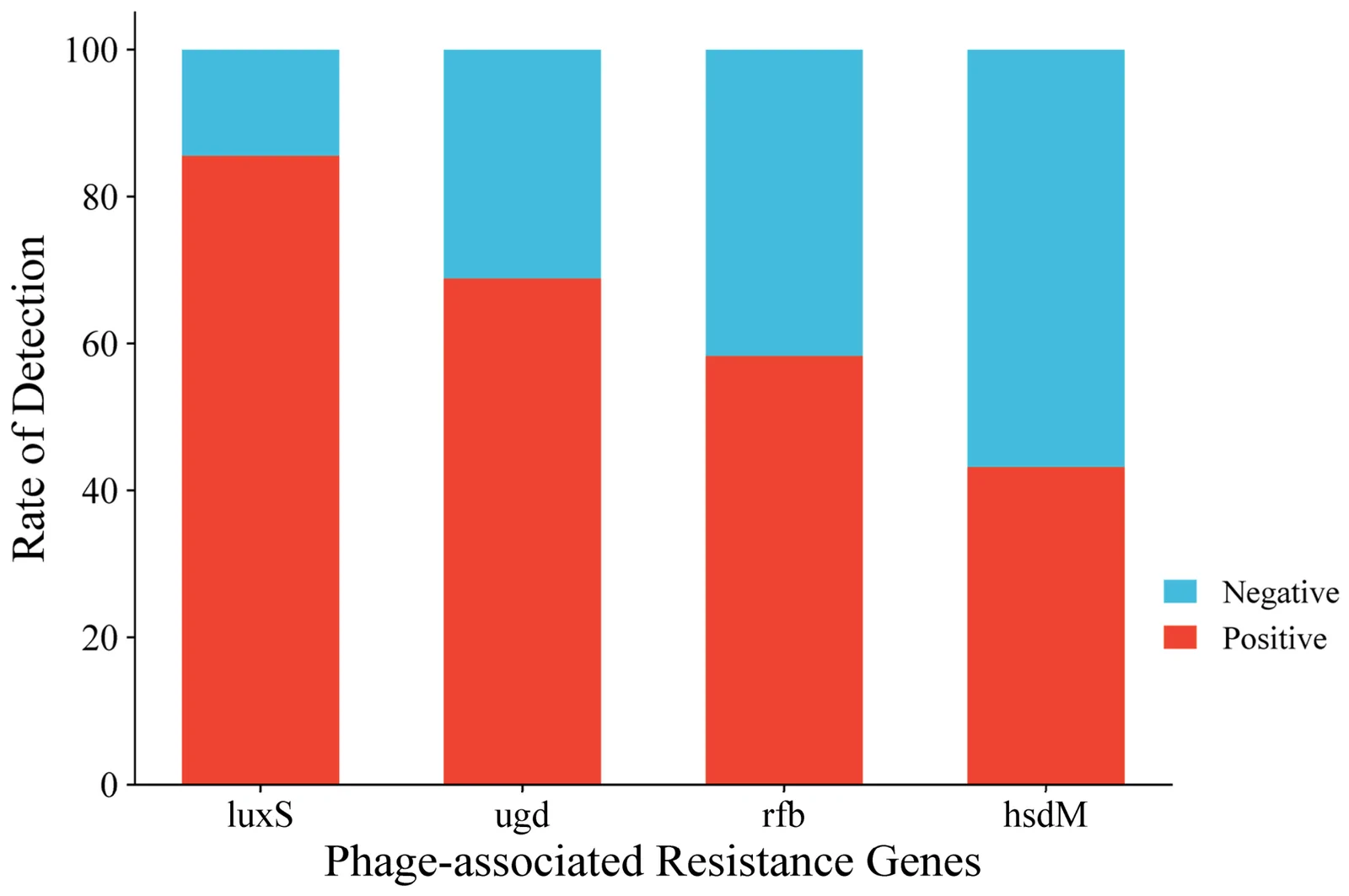
Detection rates of phage-associated resistance genes in *Vibrio parahaemolyticus* isolates. The figure shows the detection rates of selected phage-associated resistance genes among *V. parahaemolyticus* isolates. The highest detection rate was observed for *luxS* (85.61%), followed by *ugd* (68.94%) and *rfb* (58.33%), indicating that these genes were widely distributed among the tested isolates. In comparison, *hsdM* showed a lower detection rate (43.18%). Overall, the findings suggest that phage-associated genetic elements are commonly present in *V. parahaemolyticus* isolates and may contribute to the persistence and dissemination of resistance-related traits.

Four bacterial genes previously reported to influence bacterial traits related to phage infection were screened among the 132 isolates. The detected genes included *luxS*, *ugd*, *rfb*, and *hsdM*. Among the bacterial genes examined, *luxS* showed the highest detection frequency 113 (85.61%). Although *luxS* is not a phage-specific resistance determinant, its role in AI-2 quorum sensing may influence bacterial behaviors relevant to phage infection, including biofilm formation, stress adaptation, and population-level physiological regulation. Therefore, the presence of *luxS* is interpreted as an indirect indicator of bacterial traits that may affect phage susceptibility rather than direct evidence of phage resistance. The *ugd* gene was detected in 91 isolates (68.94%), followed by *rfb* in 77 isolates (58.33%) and *hsdM* in 57 isolates (43.18%) (Table 6; Figure 6). These results show that several isolates carried genes associated with quorum sensing, restriction–modification activity and lipopolysaccharide biosynthesis or modification.

### 3.6. Relationship Between Phenotypic Resistance and Detected Genes

**Figure 7:**
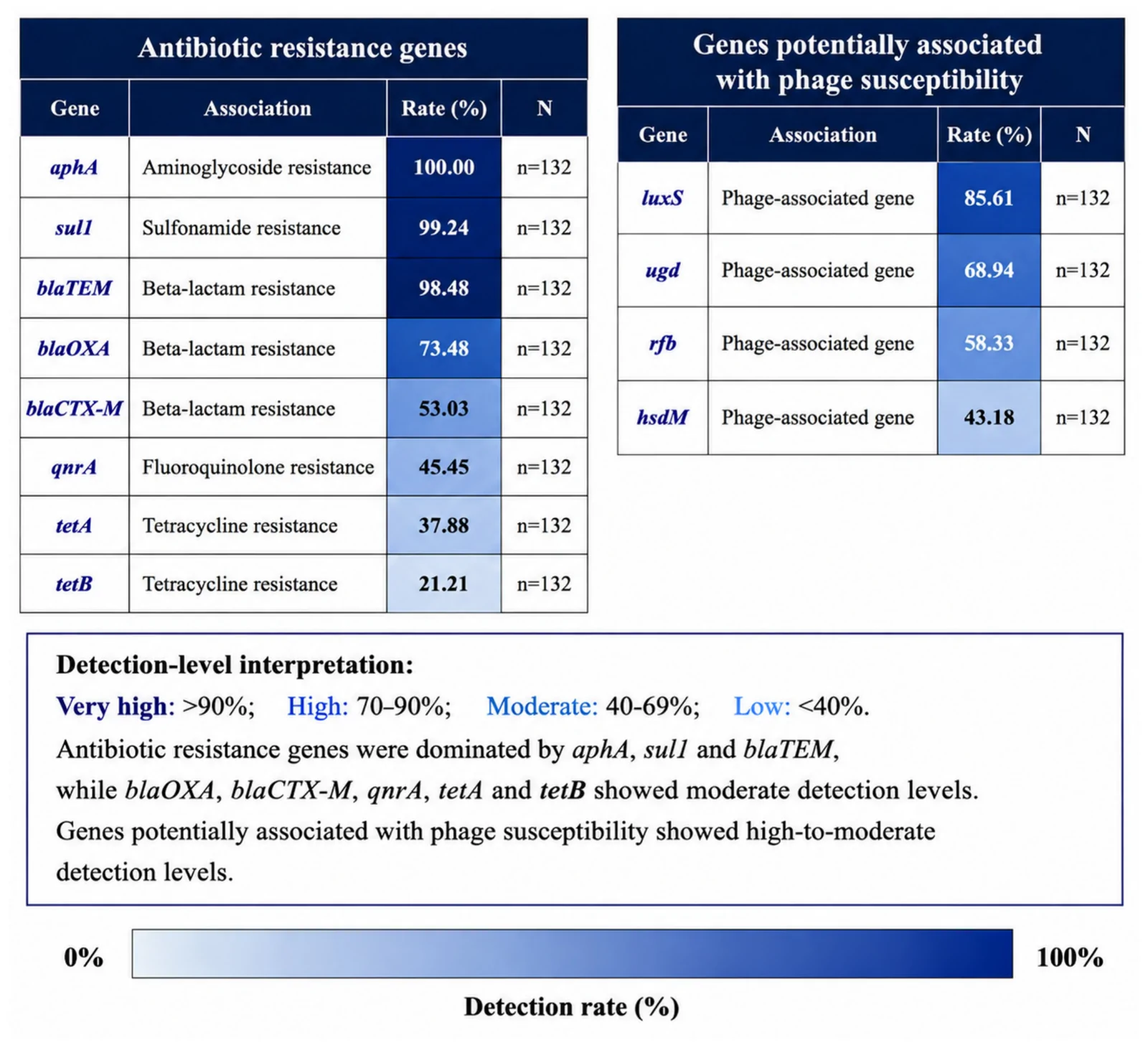
Comparative heatmap of antibiotic resistance genes and genes potentially associated with phage–host interactions detected in *Vibrio parahaemolyticus* isolates.

Comparison of phenotypic antimicrobial resistance and PCR-detected resistance genes showed both strong and partial agreement depending on the antibiotic class. Among the 132 isolates included in the molecular analysis, kanamycin resistance was high, and all isolates carried *aphA*. This indicates strong agreement between kanamycin resistance and *aphA* detection, although statistical association could not be meaningfully tested because *aphA* was present in all isolates. Similarly, sulfamethoxazole resistance corresponded closely with *sul1* detection, as sul1 was detected in 131/132 isolates (99.24%) (Table 7; Figure 7).

For β-lactam antibiotics, resistance was associated with the frequent detection of *blaTEM*, *blaOXA*, and *blaCTX-M*. However, these genes were also detected in isolates that did not show phenotypic resistance to all β-lactam antibiotics, indicating partial agreement between genotype and phenotype. This suggests that gene carriage does not always result in expressed resistance, possibly due to differences in gene expression, regulatory mechanisms, or the involvement of additional resistance determinants.

Detection of *qnrA* was only partially correlated with resistance to ciprofloxacin. *qnrA* was detected in 60 of 132 isolates (45.45%), but no statistically significant association was found between it and ciprofloxacin resistance. Among the screened tetracycline resistance determinants, *tetA* and *tetB* showed stronger phenotype–genotype agreement with a statistically significant association with tetracycline resistance. This suggests that the gene may contribute substantially to tetracycline resistance among the tested isolates.

The heatmap compares the detection rates of antibiotic resistance genes and genes potentially associated with phage–host interactions among *V. parahaemolyticus* isolates. Among the antibiotic resistance genes, *aphA* showed the highest detection rate (100%), followed by *sul1* (99.24%) and *blaTEM* (98.48%), indicating widespread carriage of aminoglycoside, sulfonamide, and β-lactam resistance determinants. *blaOXA* was also frequently detected (73.48%), while *blaCTX-M* was detected in (53.03%) of tested isolates. The fluoroquinolone resistance gene *qnrA* was detected in 45.45% of isolates, while the tetracycline resistance genes *tetA* and *tetB* were detected in 37.88% and 21.21% of isolates, respectively. Among the genes potentially associated with phage–host interactions, *luxS* showed the highest detection rate (85.61%), followed by *ugd* (68.94%), *rfb* (58.33%), and *hsdM* (43.18%). Overall, antibiotic resistance genes showed higher detection rates than most genes potentially associated with phage–host interactions, suggesting a high resistance gene burden in the isolates, while the detection of genes potentially associated with phage–host interactions may indicate possible roles in bacterial adaptation, persistence, or horizontal gene transfer.

### 3.7. Association Between Antibiotic Resistance Genes and Bacteriophage Response

Fisher’s exact test was used to evaluate the association between detected antibiotic resistance genes and reduced susceptibility to the tested bacteriophage preparations at 4 h and 5 h after phage exposure. Because multiple genes were tested at two different time points, *p*-values were adjusted using the Benjamini–Hochberg false discovery rate (FDR) correction. Several genes showed nominal associations with phage response before multiple-testing correction (Table 8). At 4 h, *blaOXA* showed an inverse association with reduced susceptibility to the tested phage preparation (OR = 0.31, 95% CI: 0.11–0.87, *p* = 0.0295), while *tetA* showed a positive association with reduced susceptibility to the tested phage preparation (OR = 3.19, 95% CI: 1.33–7.68, *p* = 0.0102). At 5 h, *blaOXA* remained inversely associated with phage resistance (OR = 0.27, 95% CI: 0.10–0.76, *p* = 0.0107), and *tetB* showed a positive association (OR = 5.00, 95% CI: 1.42–17.66, *p* = 0.0062). However, these associations did not remain statistically significant after FDR correction, indicating that they should be interpreted as exploratory trends requiring further validation. The complete association matrix, including FDR-adjusted q-values, is provided in Supplementary Table S8.

## 4. Discussion

### 4.1. Exceptional Multidrug Resistance Burden and Antibiotic-Specific Resistance Patterns in Aquaculture-Associated Vibrio parahaemolyticus: Comparison with Previous Studies

This study revealed an exceptionally high burden of antimicrobial resistance among aquaculture-associated isolates identified as *Vibrio parahaemolyticus* based on integrated phenotypic and molecular characterization recovered from shrimp farms in Zhanjiang, China, with resistance predominantly concentrated against aminoglycosides, sulfonamides, and selected β-lactam antibiotics. All isolates were sensitive to ofloxacin and levofloxacin, although resistance to kanamycin, sulfamethoxazole, cefazolin, imipenem, amoxicillin, and ampicillin was highest. Unlike a generalized resistance pattern across all antimicrobial classes, the present findings demonstrate a selective resistance profile, where certain antibiotic groups showed extensive loss of activity while others remained highly effective. Similar resistance profiles have been reported in *Vibrio* isolates from coastal rivers, shrimp farms and seafood products [36,46,47]. However, the level of resistance observed in this study, in particular the near-universal occurrence of MDR, implies that the antimicrobial resistance pressure in the aquaculture environment studied may be high.

The high level of resistance observed in this study might be attributed to the continuous antimicrobial selection pressure in aquaculture systems. Aquatic environments are regarded as important reservoirs for antimicrobial-resistant bacteria, as antibiotic residues, resistant microorganisms, and resistance determinants can enter production systems through pond management practices, sediment accumulation, animal waste discharge, agricultural runoff and human-associated contamination [48,49,50]. The interplay between water, sediment, and microbial communities creates conditions that are conducive to horizontal gene transfer, allowing the maintenance and spread of resistance determinants among bacterial populations [51,52]. Previous studies have demonstrated that aquatic ecosystems can maintain aminoglycoside and sulfonamide resistance determinants even after reductions in antimicrobial usage, suggesting long-term persistence of resistance traits in environmental bacterial communities [53,54]. This view is supported by the strong resistance observed to kanamycin and sulfamethoxazole in the present study and suggests that aquaculture environments may act as reservoirs of clinically relevant resistance genes.

The present study demonstrated one of its most important findings in the form of an extremely high prevalence of MDR among the tested isolates. Out of 132 isolates in this analysis, 131 were MDR (Figure 3), indicating that this is an almost universal characteristic among the isolates tested. This agrees with previous reports on the rising prevalence of MDR in *V. parahaemolyticus* from seafood and aquaculture environments [55,56,57]. However, the MDR prevalence observed in this study seems to be higher than that of many previously reported environmental *Vibrio* populations, suggesting that the sampled aquaculture system may represent a highly selective environment favoring the accumulation and maintenance of resistant strains. The high MDR burden observed among isolates from Zhanjiang shrimp farms raises concerns regarding the possible transmission of resistant *V. parahaemolyticus* through aquatic products and local aquaculture-associated ecosystems and underscores the need for enhanced antimicrobial stewardship in aquaculture practices.

The present analysis found antimicrobial drugs still active against these isolates, despite a broad MDR profile being observed. The preserved effectiveness towards the isolates tested was indicated by complete susceptibility to ofloxacin and levofloxacin, and high susceptibility to meropenem, chloramphenicol, doxycycline, and gentamicin (Table 3; Figure 2). We have previously demonstrated a similar variable antimicrobial response [58,59,60]. *Vibrio* is likely a function of co-induction of resistance in certain isolates by this antibiotic, perhaps a product of exposure history, local practices for antibiotics, and the genetic background of those isolates against which pathogenicity is being measured. The high resistance to certain antibiotics and the preserved susceptibility to others suggest that the development of antibiotic resistance in *V. parahaemolyticus* is more a consequence of specific selective pressures than of the general decline of antimicrobial activity. Therefore, antimicrobial susceptibility testing is of paramount importance for guiding appropriate treatment strategies and establishing efficient control measures for aquaculture-associated infections.

### 4.2. Resistance Mechanisms Associated with Detected Resistance Determinants

The identification of various antimicrobial resistance genes in *Vibrio parahaemolyticus* isolates supported the resistance phenotypes observed in this study. In agreement with the wide distribution of aminoglycoside resistance determinants among the isolates, complete detection of *aphA* (132/132 isolates) correlated with a very high phenotypic resistance to kanamycin (95.5%). Although *aphA* is typically associated with aminoglycoside modification via phosphotransferase activity, it was universally detected but not associated with gentamicin resistance in this study; thus, the expression or substrate specificity of the mechanisms responsible for resistance may differ among aminoglycosides [49,53,56]. This indicates that a resistance gene may not necessarily confer resistance to all antibiotics of the same class.

The high prevalence of *sul1* was also in accordance with the high rate of sulfamethoxazole resistance (94.7%) detected in this study (99.24%). The strong phenotype–genotype association indicates that *sul1*-mediated sulfonamide resistance is a prevalent and prominent mechanism among the examined isolates. The wide distribution of *sul1*, which is often found in mobile genetic elements such as class 1 integrons, could aid in the maintenance and acquisition of multi-resistant phenotypes [53].

The relationship between genotype and phenotype in β-lactam resistance is complex. The resistance observed in antibiotics such as ampicillin, amoxicillin, cefazolin, and other β-lactams is probably attributed to mechanisms involving β-lactamase, as indicated by the detection of *blaTEM*, *blaOXA*, and *blaCTX-M* [61,62]. However, the partial agreement observed between β-lactam resistance phenotypes and individual resistance genes indicates that resistance expression depends on multiple factors, including gene regulation, enzyme activity, membrane permeability, efflux systems, and additional resistance determinants. Therefore, the detection of a β-lactamase gene should be interpreted as evidence of resistance potential rather than a direct predictor of resistance to every β-lactam antibiotic.

Ciprofloxacin resistance also showed only partial agreement with *qnrA* detection. Although *qnrA* was identified in 45.45% of isolates, its association with ciprofloxacin resistance was not statistically significant, suggesting that additional mechanisms may contribute to quinolone resistance in the studied population (Table 5; Figure 5). These mechanisms may include mutations in quinolone target genes (*gyrA*, *gyrB*, *parC*, and *parE*), altered membrane permeability, or increased efflux activity [63,64].

For tetracycline resistance, *tetA* and *tetB* were considered relevant determinants because their gene presence alone may contribute to the resistant phenotype. In the present study, *tetA* and *tetB* showed stronger agreement with tetracycline resistance.

### 4.3. High Frequency of Reduced Phage Susceptibility and Potential Genetic Factors Associated with Phage–Host Interaction

In the present study, a large proportion of *Vibrio parahaemolyticus* isolates exhibited reduced susceptibility to phage exposure, with 93/132 isolates (70.45%) at 4 h and 90/132 isolates (68.18%) at 5 h classified as exhibiting reduced susceptibility to the tested phage preparation (Table 4). These results indicate that reduced susceptibility to phage exposure was frequently observed among the studied isolates, suggesting that a considerable proportion of this population survived phage challenge. Although reduced phage susceptibility can arise through multiple bacterial mechanisms, including receptor modification, restriction–modification systems, CRISPR-Cas defense, abortive infection systems, and biofilm-associated protection, the genetic factors identified in this study may contribute to differences in phage responsiveness among the tested isolates [65,66,67].

Among the identified genes potentially associated with phage–host interactions, *luxS* was the most abundant (85.61%), suggesting that quorum-sensing-related traits may also be implicated in bacterial persistence during phage challenge. The *luxS* gene, which is involved in the regulation of autoinducer-2 signaling and bacterial communication, influences biofilm formation, motility, and environmental stress response in *Vibrio* species. The high prevalence of *luxS* suggests that quorum-sensing-related traits may potentially influence bacterial persistence during phage challenge [65,68,69]. Biofilm formation can decrease phage efficacy by limiting penetration, increasing protection by an extracellular matrix, and generating metabolically inactive bacterial subpopulations with reduced susceptibility to phage infection [70,71,72].

The presence of *ugd* (68.94%) and *rfb* (58.33%) suggests that surface-associated processes may contribute to reduced phage susceptibility in the studied isolates. Both genes are involved in lipopolysaccharide (LPS) synthesis or modification, and LPS structures are widely used as bacteriophage adsorption receptors [73,74,75]. As a result, the comparatively high occurrence of these genes suggests that alterations to bacterial surface structures may influence phage adhesion and infection efficiency [65,76]. However, because this study only examined gene presence, direct alteration of LPS receptors cannot be established. More research with LPS profiling, adsorption tests, and whole-genome sequencing is needed to validate this process.

The occurrence of *hsdM* in 43.18% of the isolates indicates the involvement of restriction–modification defense mechanisms. Restriction–modification systems can protect bacterial cells by detecting and degrading invading DNA with aberrant methylation [77]. This would provide an additional barrier to phage replication in isolates containing *hsdM*, although functional validation is required to demonstrate that the expression of this system contributes to reduced phage susceptibility in these strains.

Several resistance genes showed nominal associations with phage response; however, these associations did not remain significant after multiple-testing correction. Therefore, these findings should be considered exploratory observations rather than evidence of direct genetic determinants of phage susceptibility. Before multiple-testing correction, *blaOXA* showed inverse associations with reduced susceptibility at 4 and 5 h, while *tetA* and *tetB* showed positive associations at the respective time points (Table 8). These observations represent exploratory correlations between antibiotic resistance gene profiles and responses to the tested phage preparations. Alternatively, they may arise from shared genomic backgrounds, plasmid carriers, cell coat traits, or stress responses that impact both antimicrobial and phage susceptibility. Additionally, because the isolated bacteriophages originated from specific sampling locations and were evaluated against the corresponding *V. parahaemolyticus* isolates from those locations, complete separation of phage-specific effects from environmental origin effects was not possible in the present study. Therefore, differences in susceptibility patterns may reflect contributions from phage characteristics, bacterial population structure, environmental variation, or their interactions. Future studies involving larger phage collections, replicated phage–host combinations across multiple sampling locations, and statistical approaches capable of incorporating phage identity and environmental origin will provide further resolution of these effects.

The observed trends involving *tetA* and *tetB* at 5 h suggest that tetracycline resistance genetic backgrounds may warrant further investigation in relation to reduced phage susceptibility; however, additional experimental validation is required to determine whether these relationships reflect direct biological mechanisms or indirect evolutionary associations.

It should be noted that the genes investigated in this study represent bacterial determinants, genes potentially associated with phage–host interactions rather than definitive phage resistance genes. Gene presence alone does not confirm functional contribution to phage resistance. Future studies integrating transcriptomic analysis, whole-genome sequencing, adsorption assays, and gene knockout approaches are required to clarify the mechanistic contribution of these determinants.

Although efficiency of plating (EOP) provides a quantitative measurement of productive phage infection and replication efficiency on different bacterial hosts, the objective of the present study was to evaluate comparative phage susceptibility patterns and growth inhibition responses among a collection of environmental *Vibrio parahaemolyticus* isolates obtained from different aquaculture sampling locations. Therefore, a microplate-based growth inhibition approach was applied as a high-throughput quantitative method to evaluate phage-mediated suppression of bacterial growth across numerous isolates. This approach enabled comparative assessment of phage responsiveness within the tested population and supported the identification of isolates exhibiting reduced susceptibility to phage exposure.

### 4.4. Potential Application of Phage-Based Control Strategies in Aquaculture

The prevalence of multidrug-resistant *Vibrio parahaemolyticus* reported in this study indicates the need for alternative measures to control pathogens in shrimp farming. Individually, the wide-ranging antimicrobial resistance of isolates and decreased phage sensitivity observed in exposure trials indicate that antibiotic control alone may not sufficiently regulate resistant *V. parahaemolyticus* populations in aquaculture.

Although phage therapy holds great promise because it is specific targeted bacteria and has minimal effects on beneficial microbial communities, the large proportion of phage-resistant isolates in this study highlights a central theme: the effective application of phages requires the identification and careful selection of active phages for use against defined bacterial populations. Phage cocktails or integrated methods that merge phage use with improved biosecurity, water quality, and antimicrobial stewardship may represent more sustainable and long-term solutions for regulating *V. parahaemolyticus* in aquaculture systems.

### 4.5. Limitations of the Study

Several limitations should be considered when interpreting the findings of this study. First, the phage susceptibility analysis was performed using the isolated phage preparations against the collected *V. parahaemolyticus* isolates, and therefore the observed susceptibility patterns may not represent all environmental populations of *V. parahaemolyticus*. Second, although several bacterial genes potentially involved in phage–host interactions were detected, their functional roles were not experimentally validated. Therefore, these genes should be considered candidate factors associated with phage susceptibility rather than confirmed resistance determinants. Third, further characterization of isolated phages, including complete genome sequencing and morphological analysis, would provide additional insights into phage taxonomy and host interaction mechanisms. Future studies involving larger collections of isolates and multiple phage preparations are required to validate and extend these findings.

## 5. Conclusions

This study revealed a high prevalence of antibiotic resistance among *V. parahaemolyticus* isolates recovered from shrimp aquaculture farms in Zhanjiang, Guangdong Province, China, and less evident multidrug resistance was reported in the isolates of this population. Although many drugs remain effective, the high-resistance profile suggests aquaculture scenarios as potential main sinks of resistant *V. parahaemolyticus* populations. Molecular characterization confirmed the widespread presence of antimicrobial resistance determinants, with a strong phenotype–genotype correlation for specific resistance traits. Nevertheless, the observed partial concordance in some resistance phenotypes with the identified genes suggests that antimicrobial resistance in *V. parahaemolyticus* is likely driven by a mixture of genetic and regulatory variables, as opposed to unique resistance determinants. Moreover, the study revealed a wide distribution of reduced phage susceptibility among isolates, consistent with the extensive loss of host ability across broader geographic regions, indicating that regional or even local-scale evaluations of phage-based control methods must prove effective prior to use in phage-applicable systems. Antimicrobial resistance and lower phage susceptibility co-existed, indicating the necessity of comprehensive controls in aquaculture systems that consider antimicrobial management, surveillance for resistance, and improvement in the selection of the most potent phages for the sustainable control of *V. parahaemolyticus*.

## Figures and Tables

**Table 1:** Geographical coordinates and sampling locations of the study sites in Zhanjiang City, Guangdong Province, China.

Site Code	Sampling Location	Latitude (N)	Longitude (E)
LVP	Lindong Village	21.11754	110.33633
XVP	Xinliao Town	20.62073	110.44837
GVP	Gaoliao Village	21.18069	110.44373
WVP	Wensheng Village	21.06749	110.33641
HVP	Huguang Town	21.10984	110.30651
YVP	Yingzai Town	21.46756	109.91341
SVP	Donghai Dao (Donghai Island)	20.99961	110.40377

**Table 2:** Distribution of collected samples and recovery of confirmed *Vibrio parahaemolyticus* isolates from different aquaculture sample types and sites.

Sample Type	Sampling Site	Number of Samples Collected	Presumptive *Vibrio* Isolates Obtained	Confirmed *V. parahaemolyticus* Isolates	Confirmation Rate Among Presumptive Isolates (%)
Sediment	HVP	11	33	20	60.6
	LVP	9	27	20	74.1
	XVP	13	39	20	51.3
Total sediment		33	99	60	60.6
Water	WVP	16	48	20	41.7
	YVP	14	42	20	47.6
	GVP	12	36	16	44.4
Total water		42	126	56	44.4
Shrimp intestine	SVP	14	42	16	38.1
Total shrimp intestine		14	42	16	38.1
Overall		89	267	132	49.4

**Table 3:** Antibiotic susceptibility profile of 132 *Vibrio parahaemolyticus* isolates.

Antibiotic	Susceptible n (%)	Intermediate n (%)	Resistant n (%)
Kanamycin	0 (0.0)	6 (4.5)	126 (95.5)
Gentamicin	119 (90.2)	13 (9.8)	0 (0.0)
Ampicillin	44 (33.3)	2 (1.5)	86 (65.2)
Amoxicillin	37 (28.0)	2 (1.5)	93 (70.5)
Ceftazidime	74 (56.1)	27 (20.5)	31 (23.5)
Cefazolin	11 (8.3)	17 (12.9)	104 (78.8)
Cefuroxime sodium salt	31 (23.5)	56 (42.4)	45 (34.1)
Cefotaxime	49 (37.1)	44 (33.3)	39 (29.5)
Cefoxitin	90 (68.2)	35 (26.5)	7 (5.3)
Imipenem	0 (0.0)	28 (21.2)	104 (78.8)
Meropenem	131 (99.2)	1 (0.8)	0 (0.0)
Ciprofloxacin	10 (7.6)	58 (43.9)	64 (48.5)
Ofloxacin	132 (100.0)	0 (0.0)	0 (0.0)
Levofloxacin	132 (100.0)	0 (0.0)	0 (0.0)
Tetracycline	66 (50.0)	22 (16.7)	44 (33.3)
Doxycycline	125 (94.7)	0 (0.0)	7 (5.3)
Sulfamethoxazole	0 (0.0)	7 (5.3)	125 (94.7)
Chloramphenicol	128 (97.0)	4 (3.0)	0 (0.0)

**Table 4:** Bacteriophage susceptibility profile of *Vibrio parahaemolyticus* isolates based on isolate-majority classification.

Time Point	Total Isolates	Resistant n (%)	Susceptible n (%)
4 h	132	93 (70.45)	39 (29.55)
5 h	132	90 (68.18)	42 (31.82)

**Table 5:** Detection frequency of antibiotic resistance genes among 132 *Vibrio parahaemolyticus* isolates.

Gene	Associated Resistance Type	Positive Isolates n (%)
*aphA*	Aminoglycoside resistance	132 (100.00)
*sul1*	Sulfonamide resistance	131 (99.24)
*blaTEM*	β-lactam resistance	130 (98.48)
*blaOXA*	β-lactam resistance	97 (73.48)
*blaCTX-M*	β-lactam resistance	70 (53.03)
*qnrA*	Quinolone resistance	60 (45.45)
*tetA*	Tetracycline resistance	50 (37.88)
*tetB*	Tetracycline resistance	28 (21.21)

**Table 6:** Detection frequency of phage-associated resistance genes among 132 *Vibrio parahaemolyticus* isolates.

Gene	Possible Function	Positive Genes Detected n	Number of Strains Tested (N)	Detection (%)
*luxS*	Quorum sensing regulation	113	132	85.61
*ugd*	LPS biosynthesis/modification	91	132	68.94
*rfb*	O-antigen/LPS biosynthesis	77	132	58.33
*hsdM*	Restriction–modification system	57	132	43.18

**Table 7:** Phenotype–genotype agreement between antimicrobial resistance and detected resistance genes in *Vibrio parahaemolyticus* isolates.

Antibiotic	Gene	Agreement (%)	Kappa	OR	Fisher’s *p*-Value	Interpretation
Kanamycin	*aphA*	95.45	0.00	19.46	1.000	High co-occurrence; no gene-negative isolates
Sulfamethoxazole	*sul1*	93.94	−0.01	5.53	1.000	High co-occurrence; weak statistical agreement
Ceftazidime	*blaCTX-M*	50.89	0.13	2.57	0.0705	Weak trend
Ciprofloxacin	*qnrA*	57.58	0.15	1.85	0.1153	Partial agreement
Tetracycline	*tetA*	60.61	0.15	1.90	0.0936	Weak trend
Tetracycline	*tetB*	75.76	0.41	9.48	<0.001	Significant/moderate agreement

**Table 8:** Nominal associations between antibiotic resistance genes and reduced susceptibility to the tested bacteriophage preparations in *Vibrio parahaemolyticus* isolates before multiple-testing correction.

Endpoint	Gene	OR	95% CI	Fisher *p*-Value	FDR-Adjusted q-Value	Interpretation
4 h	*blaOXA*	0.31	0.11–0.87	0.0295	0.118	Nominal inverse association
4 h	*tetA*	3.19	1.33–7.68	0.0102	0.057	Nominal positive association
5 h	*blaOXA*	0.27	0.10–0.76	0.0107	0.057	Nominal inverse association
5 h	*tetB*	5.00	1.42–17.66	0.0062	0.057	Nominal positive association

## Data Availability

The original contributions presented in this study are included in the article/Supplementary Materials. Further inquiries can be directed to the corresponding authors.

## Supplementary Materials

The following supporting information can be downloaded at https://www.mdpi.com/article/10.3390/microorganisms14092004/s1, Supplementary Dataset S1: Isolate-level dataset for antimicrobial susceptibility, resistance gene detection, and bacteriophage response in *Vibrio parahaemolyticus* isolates; Supplementary Table S1: Preparation information and two-fold serial dilution ranges of the 18 antibiotics used for minimum inhibitory concentration (MIC) determination of Vibrio parahaemolyticus isolates; Supplementary Table S2: Raw minimum inhibitory concentration (MIC) values of 18 antibiotics against 132 Vibrio parahaemolyticus isolates recovered from shrimp aquaculture farms in Zhanjiang, China; Supplementary Table S2A: MIC breakpoint criteria used for antimicrobial susceptibility interpretation of Vibrio parahaemolyticus isolates; Supplementary Table S3: Reported environmental conditions influencing bacteriophage activity against Vibrio parahaemolyticus; Supplementary Table S4: Detection of phage susceptibility strains in Vibrio parahaemolyticus isolates; Supplementary Table S5: Primer sequences, target genes, and expected amplicon sizes used for PCR detection of antimicrobial resistance and phage-host interaction-associated genes in Vibrio parahaemolyticus isolates; Supplementary Table S6: Replicate-Level Bacteriophage Response of Vibrio parahaemolyticus Isolates at 4 h and 5 h; Supplementary Table S7: Site-specific distribution of phage-resistant and phage-susceptible Vibrio parahaemolyticus isolates at 4 h and 5 h incubation periods; Supplementary Table S8: Full Association Analysis Between Antibiotic Resistance Genes and Reduced Susceptibility to the Tested Bacteriophage Preparations in Vibrio parahaemolyticus Isolates; Supplementary Figure S1: Initial spot assay screening of phage activity against *Vibrio parahaemolyticus* isolates.
