# Supplementary material for: Antimicrobial Resistance Profiles, Resistance Genes, and Their Association with Bacteriophage Susceptibility Among Vibrio parahaemolyticus Isolates from Zhanjiang Shrimp Aquaculture Farms
Source: Microorganisms. 2026 Sep 9;14(9):2004. doi: 10.3390/microorganisms14092004 (PMC13608986; doi:10.3390/microorganisms14092004)
Supplement: Supplementary file 1 [file microorganisms-14-02004-s001.zip › Supplementary Figure S1.pdf]

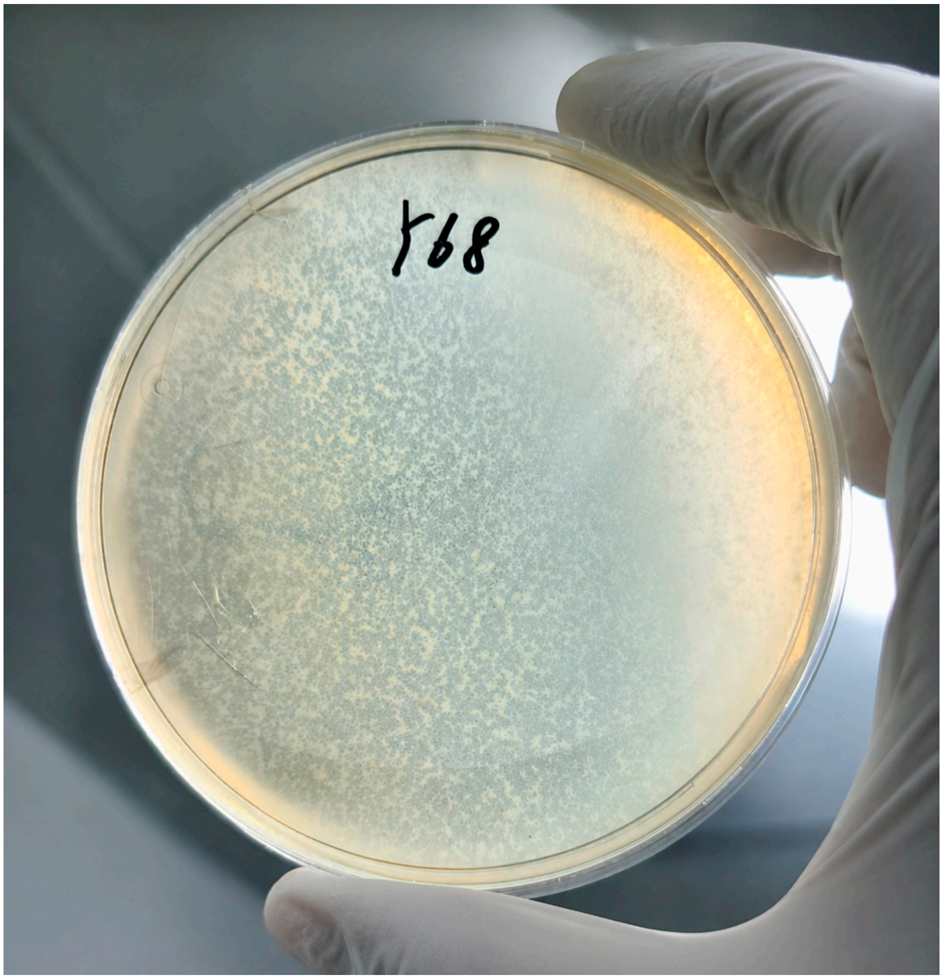

Supplementary Figure S1. Initial spot assay screening of phage activity against *Vibrio parahaemolyticus* isolates.
