# Supplementary material for: Antimicrobial Resistance Profiles, Resistance Genes, and Their Association with Bacteriophage Susceptibility Among Vibrio parahaemolyticus Isolates from Zhanjiang Shrimp Aquaculture Farms
Source: Microorganisms. 2026 Sep 9;14(9):2004. doi: 10.3390/microorganisms14092004 (PMC13608986; doi:10.3390/microorganisms14092004)
Supplement: Supplementary file 1 [file microorganisms-14-02004-s001.zip › Supplementary Table S3.pdf]

Supplementary Table S3. Reported environmental conditions influencing bacteriophage activity against *Vibrio parahaemolyticus*.

**Parameter**	**Tested condition reported in the experiment**	**Expected phage response**	**Interpretation**
Temperature	37 °C	High phage activity	Optimal infection range
pH	pH 7	Maintained infectivity	Suitable physiological range
NaCl	3.5% NaCl	Maintained activity	Compatible with marine *Vibrio* environment
