# Supplementary material for: Antimicrobial Resistance Profiles, Resistance Genes, and Their Association with Bacteriophage Susceptibility Among Vibrio parahaemolyticus Isolates from Zhanjiang Shrimp Aquaculture Farms
Source: Microorganisms. 2026 Sep 9;14(9):2004. doi: 10.3390/microorganisms14092004 (PMC13608986; doi:10.3390/microorganisms14092004)
Supplement: Supplementary file 1 [file microorganisms-14-02004-s001.zip › Supplementary Table S4.pdf]

Supplementary Table S4. Detection of phage susceptibility strains in *Vibrio parahaemolyticus* isolates

Strain (Huguang Town) (HVP)	Replicate	OD_{600} Control (4 h)	OD_{600} + Phage (4 h)	OD2/OD1 (4 h)	Effect at 4 h	OD_{600} Control (5 h)	OD_{600} + Phage (5 h)	OD2/OD1 (5 h)	Effect at 5 h
HVP1	1	0.895	0.953	1.065	Resistant	0.753	0.983	1.305	Resistant
	2	0.895	0.988	1.104	Resistant	0.753	0.872	1.158	Resistant
	3	0.895	0.853	0.953	Susceptibility	0.753	0.911	1.210	Resistant
HVP2	1	0.521	0.589	1.131	Resistant	0.626	0.775	1.238	Resistant
	2	0.521	0.723	1.388	Resistant	0.626	0.762	1.217	Resistant
	3	0.521	0.621	1.192	Resistant	0.626	0.789	1.260	Resistant
HVP3	1	0.683	0.989	1.448	Resistant	0.764	0.863	1.130	Resistant
	2	0.683	0.613	0.898	Susceptibility	0.764	0.912	1.194	Resistant
	3	0.683	0.756	1.107	Resistant	0.764	0.886	1.160	Resistant
HVP4	1	0.578	0.528	0.913	Susceptibility	0.456	0.415	0.910	Susceptibility
	2	0.578	0.734	1.270	Resistant	0.456	0.362	0.794	Susceptibility
	3	0.578	0.682	1.180	Resistant	0.456	0.618	1.355	Resistant
HVP5	1	0.856	1.325	1.548	Resistant	0.683	0.735	1.076	Resistant
	2	0.856	1.023	1.195	Resistant	0.683	0.821	1.202	Resistant
	3	0.856	1.134	1.325	Resistant	0.683	0.934	1.367	Resistant
HVP6	1	0.532	0.579	1.088	Resistant	0.364	0.316	0.868	Susceptibility
	2	0.532	0.585	1.100	Resistant	0.364	0.411	1.129	Resistant
	3	0.532	0.684	1.286	Resistant	0.364	0.783	2.151	Resistant
HVP7	1	0.744	0.712	0.957	Susceptibility	0.837	0.725	0.866	Susceptibility
	2	0.744	0.873	1.173	Resistant	0.837	0.863	1.031	Resistant
	3	0.744	0.926	1.245	Resistant	0.837	0.921	1.100	Resistant
HVP8	1	0.566	0.634	1.120	Resistant	0.421	0.573	1.361	Resistant
	2	0.566	0.688	1.216	Resistant	0.421	0.511	1.214	Resistant
	3	0.566	0.714	1.261	Resistant	0.421	0.638	1.515	Resistant
HVP9	1	0.683	0.849	1.243	Resistant	0.579	0.612	1.057	Resistant
	2	0.683	0.849	1.243	Resistant	0.579	0.784	1.354	Resistant
	3	0.683	0.849	1.243	Resistant	0.579	0.856	1.478	Resistant
HVP10	1	0.477	0.576	1.208	Resistant	0.554	0.996	1.798	Resistant
	2	0.477	0.553	1.159	Resistant	0.554	0.812	1.466	Resistant
	3	0.477	0.616	1.291	Resistant	0.554	0.873	1.576	Resistant
HVP11	1	0.742	0.853	1.150	Resistant	0.542	0.667	1.231	Resistant

	2	0.742	0.886	1.194	Resistant	0.542	0.625	1.153	Resistant
	3	0.742	0.957	1.290	Resistant	0.542	0.678	1.251	Resistant
HVP12	1	0.427	0.614	1.438	Resistant	0.649	0.836	1.288	Resistant
	2	0.427	0.792	1.855	Resistant	0.649	0.863	1.330	Resistant
	3	0.427	0.731	1.712	Resistant	0.649	0.782	1.205	Resistant
HVP13	1	0.643	0.886	1.378	Resistant	0.445	0.684	1.537	Resistant
	2	0.643	0.984	1.530	Resistant	0.445	0.959	2.155	Resistant
	3	0.643	0.752	1.170	Resistant	0.445	0.797	1.791	Resistant
HVP14	1	0.583	0.532	0.913	Susceptibility	0.532	0.463	0.870	Susceptibility
	2	0.583	0.539	0.925	Susceptibility	0.532	0.582	1.094	Resistant
	3	0.583	0.815	1.398	Resistant	0.532	0.654	1.229	Resistant
HVP15	1	0.374	0.257	0.687	Susceptibility	0.418	0.386	0.923	Susceptibility
	2	0.374	0.416	1.112	Resistant	0.418	0.426	1.019	Resistant
	3	0.374	0.522	1.396	Resistant	0.418	0.667	1.596	Resistant
HVP16	1	0.562	0.732	1.302	Resistant	0.375	0.414	1.104	Resistant
	2	0.562	0.941	1.674	Resistant	0.375	0.551	1.469	Resistant
	3	0.562	0.866	1.541	Resistant	0.375	0.739	1.971	Resistant
HVP17	1	0.623	0.878	1.409	Resistant	0.268	0.481	1.795	Resistant
	2	0.623	0.611	0.981	Susceptibility	0.473	0.575	1.216	Resistant
	3	0.623	0.737	1.183	Resistant	0.473	0.863	1.825	Resistant
HVP18	1	0.537	0.753	1.402	Resistant	0.542	0.826	1.524	Resistant
	2	0.537	0.883	1.644	Resistant	0.542	0.642	1.185	Resistant
	3	0.537	0.892	1.661	Resistant	0.542	0.721	1.330	Resistant
HVP19	1	0.485	0.396	0.816	Susceptibility	0.382	0.788	2.063	Resistant
	2	0.485	0.746	1.538	Resistant	0.382	0.842	2.204	Resistant
	3	0.485	0.993	2.047	Resistant	0.382	0.868	2.272	Resistant
HVP20	1	0.643	0.518	0.806	Susceptibility	0.527	0.853	1.619	Resistant
	2	0.643	0.692	1.076	Resistant	0.527	0.892	1.693	Resistant
	3	0.643	0.727	1.131	Resistant	0.527	0.744	1.412	Resistant

Strain (Lindong Village) (LVP)	Replicate	OD_{600} Control (4 h)	OD_{600} + Phage (4 h)	OD2/OD1 (4 h)	Effect at 4 h	OD_{600} Control (5 h)	OD_{600} + Phage (5 h)	OD2/OD1 (5 h)	Effect at 5 h
LVP3	1	0.732	0.619	0.846	Susceptibility	0.576	0.486	0.844	Susceptibility
	2	0.732	0.542	0.740	Susceptibility	0.576	0.413	0.717	Susceptibility
	3	0.732	0.753	1.029	Resistant	0.576	0.346	0.601	Susceptibility
LVP5	1	0.467	0.385	0.824	Susceptibility	0.357	0.243	0.681	Susceptibility
	2	0.467	0.423	0.906	Susceptibility	0.357	0.165	0.462	Susceptibility
	3	0.467	0.321	0.687	Susceptibility	0.357	0.312	0.874	Susceptibility
LVP7	1	0.528	0.335	0.634	Susceptibility	0.475	0.487	1.025	Resistant
	2	0.528	0.402	0.761	Susceptibility	0.475	0.522	1.099	Resistant
	3	0.528	0.212	0.402	Susceptibility	0.475	0.365	0.768	Susceptibility
LVP9	1	0.398	0.528	1.327	Resistant	0.637	0.525	0.824	Susceptibility
	2	0.398	0.635	1.595	Resistant	0.637	0.605	0.950	Susceptibility
	3	0.398	0.314	0.789	Susceptibility	0.637	0.794	1.246	Resistant
LVP11	1	0.583	0.683	1.172	Resistant	0.458	0.576	1.258	Resistant
	2	0.583	0.711	1.220	Resistant	0.458	0.531	1.159	Resistant
	3	0.583	0.732	1.256	Resistant	0.458	0.384	0.838	Susceptibility
LVP12	1	0.376	0.326	0.867	Susceptibility	0.475	0.497	1.046	Resistant
	2	0.376	0.315	0.838	Susceptibility	0.475	0.426	0.897	Susceptibility
	3	0.376	0.416	1.106	Resistant	0.475	0.383	0.806	Susceptibility
LVP14	1	0.487	0.573	1.177	Resistant	0.521	0.421	0.808	Susceptibility
	2	0.487	0.582	1.195	Resistant	0.521	0.346	0.664	Susceptibility
	3	0.487	0.352	0.723	Susceptibility	0.521	0.511	0.981	Susceptibility
LVP15	1	0.746	0.676	0.906	Susceptibility	0.533	0.683	1.281	Resistant
	2	0.746	0.947	1.269	Resistant	0.533	0.753	1.413	Resistant
	3	0.746	0.653	0.875	Susceptibility	0.533	0.238	0.447	Susceptibility
LVP16	1	0.638	0.542	0.850	Resistant	0.472	0.554	1.174	Resistant
	2	0.638	0.519	0.813	Resistant	0.472	0.257	0.544	Susceptibility
	3	0.638	0.745	1.168	Susceptibility	0.472	0.375	0.794	Susceptibility
LVP19	1	0.597	0.672	1.126	Resistant	0.482	0.511	1.060	Resistant
	2	0.597	0.596	0.998	Susceptibility	0.482	0.352	0.730	Susceptibility
	3	0.597	0.616	1.032	Resistant	0.482	0.406	0.842	Susceptibility
LVP20	1	0.369	0.346	0.938	Susceptibility	0.463	0.253	0.546	Susceptibility
	2	0.369	0.254	0.688	Susceptibility	0.463	0.385	0.832	Susceptibility
	3	0.369	0.352	0.954	Susceptibility	0.463	0.573	1.238	Resistant

LVP21	1	0.529	0.352	0.665	Susceptibility	0.477	0.442	0.927	Susceptibility
	2	0.529	0.472	0.892	Susceptibility	0.477	0.433	0.908	Susceptibility
	3	0.529	0.513	0.970	Susceptibility	0.477	0.424	0.889	Susceptibility
LVP24	1	0.445	0.367	0.825	Susceptibility	0.567	0.748	1.319	Resistant
	2	0.445	0.422	0.948	Susceptibility	0.567	0.524	0.924	Susceptibility
	3	0.445	0.365	0.820	Susceptibility	0.567	0.467	0.824	Susceptibility
LVP27	1	0.366	0.238	0.650	Susceptibility	0.387	0.256	0.661	Susceptibility
	2	0.366	0.234	0.639	Susceptibility	0.387	0.582	1.504	Resistant
	3	0.366	0.295	0.806	Susceptibility	0.387	0.332	0.858	Susceptibility
LVP30	1	0.476	0.553	1.162	Resistant	0.639	0.587	0.919	Susceptibility
	2	0.476	0.336	0.706	Susceptibility	0.639	0.847	1.326	Resistant
	3	0.476	0.232	0.487	Susceptibility	0.639	0.578	0.905	Susceptibility
LVP31	1	0.563	0.337	0.599	Susceptibility	0.682	0.749	1.098	Resistant
	2	0.563	0.452	0.803	Susceptibility	0.682	0.634	0.930	Susceptibility
	3	0.563	0.426	0.757	Susceptibility	0.682	0.587	0.861	Susceptibility
LVP32	1	0.427	0.642	1.504	Resistant	0.487	0.381	0.782	Susceptibility
	2	0.427	0.517	1.211	Resistant	0.487	0.612	1.257	Resistant
	3	0.427	0.337	0.789	Susceptibility	0.487	0.743	1.526	Resistant
LVP33	1	0.622	0.586	0.942	Susceptibility	0.683	0.703	1.029	Resistant
	2	0.622	0.597	0.960	Susceptibility	0.683	0.563	0.824	Susceptibility
	3	0.622	0.614	0.987	Susceptibility	0.683	0.462	0.676	Susceptibility
LVP34	1	0.567	0.639	1.127	Resistant	0.673	0.852	1.266	Resistant
	2	0.567	0.596	1.051	Resistant	0.673	0.971	1.443	Resistant
	3	0.567	0.843	1.487	Resistant	0.673	0.768	1.141	Resistant
LVP35	1	0.377	0.358	0.950	Susceptibility	0.428	0.368	0.860	Susceptibility
	2	0.377	0.242	0.642	Susceptibility	0.428	0.269	0.629	Susceptibility
	3	0.377	0.229	0.607	Susceptibility	0.428	0.381	0.890	Susceptibility

Strain (Wensheng Village) (WVP)	Replicate	OD_{600} Control (4 h)	OD_{600} + Phage (4 h)	OD2/OD1 (4 h)	Effect at 4 h	OD_{600} Control (5 h)	OD_{600} + Phage (5 h)	OD2/OD1 (5 h)	Effect at 5 h
	1	0.839	0.799	0.952	Susceptibility	0.957	0.937	0.979	Susceptibility
WVP4	2	0.839	0.913	1.088	Resistant	0.957	0.993	1.038	Resistant
	3	0.839	0.882	1.051	Resistant	0.957	0.963	1.006	Resistant
	1	0.661	0.643	0.973	Susceptibility	0.784	0.721	0.920	Susceptibility
WVP5	2	0.661	0.681	1.030	Resistant	0.784	0.731	0.932	Susceptibility
	3	0.661	0.628	0.950	Susceptibility	0.784	0.812	1.036	Resistant
	1	0.657	0.974	1.482	Resistant	0.807	0.917	1.136	Resistant
WVP7	2	0.657	0.997	1.518	Resistant	0.807	0.871	1.079	Resistant
	3	0.657	0.872	1.327	Resistant	0.807	0.968	1.200	Resistant
	1	0.714	0.781	1.094	Resistant	0.731	0.556	0.761	Susceptibility
WVP10	2	0.714	0.812	1.137	Resistant	0.731	0.692	0.947	Susceptibility
	3	0.714	0.932	1.305	Resistant	0.731	0.786	1.075	Resistant
	1	0.576	1.131	1.964	Resistant	0.738	0.794	1.076	Resistant
WVP14	2	0.576	1.003	1.741	Resistant	0.738	0.772	1.046	Resistant
	3	0.576	1.361	2.363	Resistant	0.738	0.781	1.058	Resistant
	1	1.661	1.146	0.690	Susceptibility	1.206	1.224	1.015	Resistant
WVP15	2	1.661	1.518	0.914	Susceptibility	1.206	1.364	1.131	Resistant
	3	1.661	1.326	0.798	Susceptibility	1.206	1.291	1.070	Resistant
	1	0.694	0.787	1.134	Resistant	0.673	0.648	0.963	Susceptibility
WVP36	2	0.694	0.962	1.386	Resistant	0.673	0.549	0.816	Susceptibility
	3	0.694	0.871	1.255	Resistant	0.673	0.701	1.042	Resistant
	1	0.801	0.678	0.846	Susceptibility	0.766	0.548	0.715	Susceptibility
WVP52	2	0.801	0.893	1.115	Resistant	0.766	0.671	0.876	Susceptibility
	3	0.801	0.915	1.142	Resistant	0.766	0.599	0.782	Susceptibility
	1	0.924	1.158	1.253	Resistant	0.895	0.953	1.065	Resistant
WVP59	2	0.924	1.261	1.365	Resistant	0.895	0.981	1.096	Resistant
	3	0.924	1.311	1.419	Resistant	0.895	0.902	1.008	Resistant
	1	0.34	0.222	0.653	Susceptibility	0.44	0.32	0.727	Susceptibility
WVP60	2	0.34	0.211	0.621	Susceptibility	0.44	0.41	0.932	Susceptibility
	3	0.34	0.303	0.891	Susceptibility	0.44	0.38	0.864	Susceptibility
	1	0.949	0.914	0.963	Susceptibility	1.364	1.606	1.177	Resistant
WVP61	2	0.949	0.822	0.866	Susceptibility	1.364	1.566	1.148	Resistant
	3	0.949	0.925	0.975	Susceptibility	1.364	1.749	1.282	Resistant

	1	0.702	0.721	1.027	Resistant	0.886	0.874	0.986	Susceptibility
WVP70	2	0.702	0.768	1.094	Resistant	0.886	0.882	0.995	Susceptibility
	3	0.702	0.817	1.164	Resistant	0.886	0.877	0.990	Susceptibility
	1	1.212	1.028	0.848	Susceptibility	1.19	1.382	1.161	Resistant
WVP71	2	1.212	0.847	0.699	Susceptibility	1.19	1.221	1.026	Resistant
	3	1.212	1.011	0.834	Susceptibility	1.19	1.415	1.189	Resistant
	1	1.22	1.133	0.929	Susceptibility	1.17	1.304	1.115	Resistant
WVP73	2	1.22	1.211	0.993	Susceptibility	1.17	1.481	1.266	Resistant
	3	1.22	1.005	0.824	Susceptibility	1.17	1.296	1.108	Resistant
	1	1.272	1.139	0.895	Susceptibility	1.185	1.277	1.078	Resistant
WVP75	2	1.272	1.042	0.819	Susceptibility	1.185	1.372	1.158	Resistant
	3	1.272	1.191	0.936	Susceptibility	1.185	1.295	1.093	Resistant
	1	1.269	1.291	1.017	Resistant	1.363	1.41	1.034	Resistant
WVP84	2	1.269	1.382	1.089	Resistant	1.363	1.583	1.161	Resistant
	3	1.269	1.438	1.133	Resistant	1.363	1.447	1.062	Resistant
	1	0.565	0.509	0.901	Susceptibility	0.568	0.585	1.030	Resistant
WVP86	2	0.565	0.542	0.959	Susceptibility	0.568	0.611	1.076	Resistant
	3	0.565	0.482	0.853	Susceptibility	0.568	0.783	1.379	Resistant
	1	0.847	0.741	0.875	Susceptibility	1.283	1.028	0.801	Susceptibility
WVP94	2	0.847	0.622	0.734	Susceptibility	1.283	1.141	0.889	Susceptibility
	3	0.847	0.793	0.936	Susceptibility	1.283	1.186	0.924	Susceptibility
	1	0.38	0.477	1.255	Resistant	0.429	0.467	1.089	Resistant
WVP95	2	0.38	0.518	1.363	Resistant	0.429	0.581	1.354	Resistant
	3	0.38	0.537	1.413	Resistant	0.429	0.614	1.431	Resistant
	1	0.135	0.154	1.141	Resistant	0.159	0.198	1.245	Resistant
WVP97	2	0.135	0.218	1.615	Resistant	0.159	0.185	1.164	Resistant
	3	0.135	0.193	1.430	Resistant	0.159	0.211	1.327	Resistant

**Strain (Xinliao Town) (XVP)**	**Replicate**	**OD_{600} Control (4 h)**	**OD_{600} + Phage (4 h)**	**OD2/OD1 (4 h)**	**Effect at 4 h**	**OD_{600} Control (5 h)**	**OD_{600} + Phage (5 h)**	**OD2/OD1 (5 h)**	**Effect at 5 h**
	1	1.09	1.081	0.992	Susceptibility	1.238	1.149	0.928	Susceptibility
XVP1	2	1.09	0.849	0.779	Susceptibility	1.238	1.298	1.048	Resistant
	3	1.09	1.125	1.032	Resistant	1.238	1.025	0.828	Susceptibility
	1	0.492	0.308	0.626	Susceptibility	0.512	0.691	1.350	Resistant
XVP2	2	0.492	0.211	0.429	Susceptibility	0.512	0.582	1.137	Resistant
	3	0.492	0.472	0.959	Susceptibility	0.512	0.746	1.457	Resistant
	1	0.446	0.468	1.049	Resistant	0.476	0.166	0.349	Susceptibility
XVP3	2	0.446	0.531	1.191	Resistant	0.476	0.269	0.565	Susceptibility
	3	0.446	0.668	1.498	Resistant	0.476	0.382	0.803	Susceptibility
	1	0.452	0.33	0.730	Susceptibility	0.482	0.494	1.025	Resistant
XVP5	2	0.452	0.403	0.892	Susceptibility	0.482	0.525	1.089	Resistant
	3	0.452	0.441	0.976	Susceptibility	0.482	0.818	1.697	Resistant
	1	0.512	0.387	0.756	Susceptibility	0.502	0.474	0.944	Susceptibility
XVP8	2	0.512	0.431	0.842	Susceptibility	0.502	0.378	0.753	Susceptibility
	3	0.512	0.504	0.984	Susceptibility	0.502	0.299	0.596	Susceptibility
	1	0.201	0.282	1.403	Resistant	0.542	0.494	0.911	Susceptibility
XVP11	2	0.201	0.375	1.866	Resistant	0.542	0.511	0.943	Susceptibility
	3	0.201	0.284	1.413	Resistant	0.542	0.384	0.708	Susceptibility
	1	0.311	0.277	0.891	Susceptibility	0.483	0.459	0.950	Susceptibility
XVP12	2	0.311	0.259	0.833	Susceptibility	0.483	0.427	0.884	Susceptibility
	3	0.311	0.184	0.592	Susceptibility	0.483	0.389	0.805	Susceptibility
	1	0.263	0.308	1.171	Resistant	0.373	0.479	1.284	Resistant
XVP14	2	0.263	0.479	1.821	Resistant	0.373	0.593	1.590	Resistant
	3	0.263	0.511	1.943	Resistant	0.373	0.795	2.131	Resistant
	1	0.192	0.315	1.641	Resistant	0.256	0.502	1.961	Resistant
XVP15	2	0.192	0.285	1.484	Resistant	0.256	0.295	1.152	Resistant
	3	0.192	0.362	1.885	Resistant	0.256	0.386	1.508	Resistant
	1	0.231	0.455	1.970	Resistant	0.251	0.763	3.040	Resistant
XVP16	2	0.231	0.358	1.550	Resistant	0.251	0.682	2.717	Resistant
	3	0.231	0.413	1.788	Resistant	0.251	0.721	2.873	Resistant
	1	0.241	0.319	1.324	Resistant	0.265	0.612	2.309	Resistant
XVP17	2	0.241	0.283	1.174	Resistant	0.265	0.686	2.589	Resistant
	3	0.241	0.351	1.456	Resistant	0.265	0.598	2.257	Resistant

	1	0.192	0.315	1.641	Resistant	0.256	0.502	1.961	Resistant
XVP18	2	0.192	0.299	1.557	Resistant	0.256	0.485	1.895	Resistant
	3	0.192	0.274	1.427	Resistant	0.256	0.436	1.703	Resistant
	1	0.192	0.265	1.380	Resistant	0.248	0.491	1.980	Resistant
XVP19	2	0.192	0.315	1.641	Resistant	0.248	0.359	1.448	Resistant
	3	0.192	0.284	1.479	Resistant	0.248	0.297	1.198	Resistant
	1	0.748	0.231	0.309	Susceptibility	0.778	0.357	0.459	Susceptibility
XVP20	2	0.748	0.569	0.761	Susceptibility	0.778	0.236	0.303	Susceptibility
	3	0.748	0.286	0.382	Susceptibility	0.778	0.632	0.812	Susceptibility
	1	0.608	0.457	0.752	Susceptibility	0.592	0.486	0.821	Susceptibility
XVP21	2	0.608	0.516	0.849	Susceptibility	0.592	0.526	0.889	Susceptibility
	3	0.608	0.592	0.974	Susceptibility	0.592	0.497	0.840	Susceptibility
	1	0.618	0.33	0.534	Susceptibility	0.486	0.431	0.887	Susceptibility
XVP22	2	0.618	0.659	1.066	Resistant	0.486	0.431	0.887	Susceptibility
	3	0.618	0.528	0.854	Susceptibility	0.486	0.525	1.080	Resistant
	1	0.419	0.233	0.556	Susceptibility	0.268	0.481	1.795	Resistant
XVP24	2	0.419	0.374	0.893	Susceptibility	0.268	0.395	1.474	Resistant
	3	0.419	0.537	1.282	Resistant	0.268	0.428	1.597	Resistant
	1	0.816	0.477	0.585	Susceptibility	0.736	0.728	0.989	Susceptibility
XVP26	2	0.816	0.683	0.837	Susceptibility	0.736	0.642	0.872	Susceptibility
	3	0.816	0.792	0.971	Susceptibility	0.736	0.711	0.966	Susceptibility
	1	0.786	0.252	0.321	Susceptibility	0.596	0.288	0.483	Susceptibility
XVP28	2	0.786	0.846	1.076	Resistant	0.596	0.642	1.077	Resistant
	3	0.786	0.693	0.882	Susceptibility	0.596	0.468	0.785	Susceptibility
	1	0.255	0.288	1.129	Resistant	0.265	0.278	1.049	Resistant
XVP29	2	0.255	0.315	1.235	Resistant	0.265	0.356	1.343	Resistant
	3	0.255	0.427	1.675	Resistant	0.265	0.465	1.755	Resistant

Strain (Yingzai Town) (YVP)	Replicate	OD_{600} Control (4 h)	OD_{600} + Phage (4 h)	OD2/OD1 (4 h)	Effect at 4 h	OD_{600} Control (5 h)	OD_{600} + Phage (5 h)	OD2/OD1 (5 h)	Effect at 5 h
YVP2	1	0.638	0.523	0.820	Susceptibility	0.479	0.684	1.428	Resistant
	2	0.638	0.874	1.370	Resistant	0.479	0.591	1.234	Resistant
	3	0.638	0.787	1.234	Resistant	0.479	0.687	1.434	Resistant
YVP3	1	0.583	0.954	1.636	Resistant	0.537	0.852	1.587	Resistant
	2	0.583	0.922	1.581	Resistant	0.537	0.623	1.160	Resistant
	3	0.583	0.543	0.931	Susceptibility	0.537	0.722	1.345	Resistant
YVP15	1	0.437	0.583	1.334	Resistant	0.592	0.743	1.255	Resistant
	2	0.437	0.624	1.428	Resistant	0.592	0.682	1.152	Resistant
	3	0.437	0.682	1.561	Resistant	0.592	0.723	1.221	Resistant
YVP17	1	0.762	0.639	0.839	Susceptibility	0.332	0.467	1.407	Resistant
	2	0.762	0.892	1.171	Resistant	0.332	0.432	1.301	Resistant
	3	0.762	0.877	1.151	Resistant	0.332	0.474	1.428	Resistant
YVP18	1	0.388	0.321	0.827	Susceptibility	0.462	0.533	1.154	Resistant
	2	0.388	0.484	1.247	Resistant	0.462	0.568	1.229	Resistant
	3	0.388	0.437	1.126	Resistant	0.462	0.592	1.281	Resistant
YVP20	1	0.643	0.746	1.160	Resistant	0.527	0.732	1.389	Resistant
	2	0.643	0.755	1.174	Resistant	0.527	0.663	1.258	Resistant
	3	0.643	0.738	1.148	Resistant	0.527	0.737	1.398	Resistant
YVP21	1	0.554	0.638	1.152	Resistant	0.436	0.582	1.335	Resistant
	2	0.554	0.621	1.121	Resistant	0.436	0.593	1.360	Resistant
	3	0.554	0.668	1.206	Resistant	0.436	0.532	1.220	Resistant
YVP22	1	0.467	0.373	0.799	Susceptibility	0.368	0.463	1.258	Resistant
	2	0.467	0.526	1.126	Resistant	0.368	0.589	1.601	Resistant
	3	0.467	0.587	1.257	Resistant	0.368	0.632	1.717	Resistant
YVP27	1	0.622	0.534	0.859	Susceptibility	0.583	0.745	1.278	Resistant
	2	0.622	0.789	1.268	Resistant	0.583	0.712	1.221	Resistant
	3	0.622	0.753	1.211	Resistant	0.583	0.722	1.238	Resistant
YVP34	1	0.446	0.364	0.816	Susceptibility	0.599	0.743	1.240	Resistant
	2	0.446	0.567	1.271	Resistant	0.599	0.788	1.316	Resistant
	3	0.446	0.531	1.191	Resistant	0.599	0.729	1.217	Resistant
YVP36	1	0.583	0.719	1.233	Resistant	0.443	0.574	1.296	Resistant
	2	0.583	0.688	1.180	Resistant	0.443	0.682	1.540	Resistant
	3	0.583	0.735	1.261	Resistant	0.443	0.752	1.698	Resistant

YVP42	1	0.659	0.726	1.102	Resistant	0.655	0.856	1.307	Resistant
	2	0.659	0.731	1.109	Resistant	0.655	0.759	1.159	Resistant
	3	0.659	0.811	1.231	Resistant	0.655	0.801	1.223	Resistant
YVP46	1	0.738	0.875	1.186	Resistant	0.642	0.822	1.280	Resistant
	2	0.738	0.894	1.211	Resistant	0.642	0.855	1.332	Resistant
	3	0.738	0.946	1.282	Resistant	0.642	0.877	1.366	Resistant
YVP49	1	0.487	0.412	0.846	Susceptibility	0.386	0.465	1.205	Resistant
	2	0.487	0.648	1.331	Resistant	0.386	0.632	1.637	Resistant
	3	0.487	0.622	1.277	Resistant	0.386	0.572	1.482	Resistant
YVP50	1	0.529	0.632	1.195	Resistant	0.477	0.582	1.220	Resistant
	2	0.529	0.688	1.301	Resistant	0.477	0.611	1.281	Resistant
	3	0.529	0.728	1.376	Resistant	0.477	0.673	1.411	Resistant
YVP51	1	0.473	0.352	0.744	Susceptibility	0.386	0.573	1.484	Resistant
	2	0.473	0.684	1.446	Resistant	0.386	0.468	1.212	Resistant
	3	0.473	0.742	1.569	Resistant	0.386	0.483	1.251	Resistant
YVP61	1	0.586	0.524	0.894	Susceptibility	0.527	0.682	1.294	Resistant
	2	0.586	0.693	1.183	Resistant	0.527	0.699	1.326	Resistant
	3	0.586	0.727	1.241	Resistant	0.527	0.725	1.376	Resistant
YVP71	1	0.386	0.362	0.938	Susceptibility	0.453	0.521	1.150	Resistant
	2	0.386	0.472	1.223	Resistant	0.453	0.543	1.199	Resistant
	3	0.386	0.542	1.404	Resistant	0.453	0.649	1.433	Resistant
YVP76	1	0.482	0.382	0.793	Susceptibility	0.372	0.653	1.755	Resistant
	2	0.482	0.627	1.301	Resistant	0.372	0.536	1.441	Resistant
	3	0.482	0.641	1.330	Resistant	0.372	0.766	2.059	Resistant
YVP81	1	0.583	0.474	0.813	Susceptibility	0.735	0.826	1.124	Resistant
	2	0.583	0.621	1.065	Resistant	0.735	0.953	1.297	Resistant
	3	0.583	0.674	1.156	Resistant	0.735	0.785	1.068	Resistant

Strain (Gaoliao Village) (GVP)	Replicate	OD_{600} Control (4 h)	OD_{600} + Phage (4 h)	OD2/OD1 (4 h)	Effect at 4 h	OD_{600} Control (5 h)	OD_{600} + Phage (5 h)	OD2/OD1 (5 h)	Effect at 5 h
GVP1	1	0.130	0.499	3.838	Resistant	0.206	0.124	0.601	Susceptibility
	2	0.130	0.373	2.869	Resistant	0.206	0.332	1.611	Resistant
	3	0.130	0.285	2.192	Resistant	0.206	0.183	0.888	Susceptibility
GVP2	1	1.148	1.16	1.010	Resistant	1.171	1.399	1.194	Resistant
	2	1.148	1.43	1.245	Resistant	1.171	1.285	1.097	Resistant
	3	1.148	1.63	1.419	Resistant	1.171	1.436	1.226	Resistant
GVP3	1	0.845	1.052	1.244	Resistant	1.038	1.182	1.138	Resistant
	2	0.845	0.749	0.886	Susceptibility	1.038	1.251	1.205	Resistant
	3	0.845	1.102	1.304	Resistant	1.038	1.138	1.096	Resistant
GVP4	1	0.999	1.001	1.002	Resistant	1.112	1.377	1.238	Resistant
	2	0.999	1.214	1.215	Resistant	1.112	1.361	1.223	Resistant
	3	0.999	1.318	1.319	Resistant	1.112	1.294	1.163	Resistant
GVP5	1	1.129	1.249	1.106	Resistant	0.93	0.993	1.067	Resistant
	2	1.129	1.189	1.053	Resistant	0.93	1.042	1.120	Resistant
	3	1.129	1.342	1.188	Resistant	0.93	1.314	1.412	Resistant
GVP6	1	1.176	1.366	1.161	Resistant	0.793	0.781	0.984	Susceptibility
	2	1.176	1.275	1.084	Resistant	0.793	0.779	0.982	Susceptibility
	3	1.176	1.312	1.115	Resistant	0.793	0.869	1.095	Resistant
GVP7	1	0.883	1.031	1.167	Resistant	0.985	-0.011	-0.0112	Susceptibility
	2	0.883	1.028	1.164	Resistant	0.985	-0.018	-0.0183	Susceptibility
	3	0.883	0.976	1.105	Resistant	0.985	-0.142	-0.1446	Susceptibility
GVP8	1	0.254	0.204	0.803	Susceptibility	0.315	0.241	0.765	Susceptibility
	2	0.254	0.219	0.862	Susceptibility	0.315	0.322	1.022	Resistant
	3	0.254	0.301	1.185	Resistant	0.315	0.281	0.892	Susceptibility
GVP9	1	1.349	1.355	1.004	Resistant	0.978	0.969	0.990	Susceptibility
	2	1.349	1.379	1.022	Resistant	0.978	0.821	0.839	Susceptibility
	3	1.349	1.451	1.075	Resistant	0.978	0.893	0.913	Susceptibility
GVP10	1	0.699	0.672	0.961	Susceptibility	0.685	0.653	0.953	Susceptibility
	2	0.699	0.681	0.974	Susceptibility	0.685	0.674	0.983	Susceptibility
	3	0.699	0.593	0.848	Susceptibility	0.685	0.596	0.870	Susceptibility
GVP11	1	0.730	0.644	0.882	Susceptibility	0.909	0.901	0.991	Susceptibility
	2	0.730	0.683	0.935	Susceptibility	0.909	0.982	1.080	Resistant
	3	0.730	0.711	0.973	Susceptibility	0.909	0.879	0.966	Susceptibility

GVP12	1	0.701	0.777	1.108	Resistant	0.943	1.028	1.090	Resistant
	2	0.701	0.814	1.161	Resistant	0.943	1.271	1.347	Resistant
	3	0.701	0.793	1.131	Resistant	0.943	1.163	1.233	Resistant
GVP13	1	0.890	0.715	0.803	Susceptibility	1.003	0.949	0.946	Susceptibility
	2	0.890	0.772	0.867	Susceptibility	1.003	0.811	0.808	Susceptibility
	3	0.890	0.812	0.912	Susceptibility	1.003	0.913	0.910	Susceptibility
GVP14	1	0.637	0.666	1.045	Resistant	0.872	1.073	1.230	Resistant
	2	0.637	0.641	1.006	Resistant	0.872	0.963	1.104	Resistant
	3	0.637	0.710	1.114	Resistant	0.872	1.142	1.309	Resistant
GVP15	1	0.668	0.611	0.914	Susceptibility	0.963	0.830	0.861	Susceptibility
	2	0.668	0.636	0.952	Susceptibility	0.963	0.941	0.977	Susceptibility
	3	0.668	0.712	1.065	Resistant	0.963	0.889	0.923	Susceptibility
GVP16	1	0.875	0.947	1.082	Resistant	1.269	1.317	1.037	Resistant
	2	0.875	0.961	1.098	Resistant	1.269	1.379	1.086	Resistant
	3	0.875	1.052	1.202	Resistant	1.269	1.461	1.151	Resistant

**Strain Donghai Dao (Shrimp) (SVP)**	**Replicate**	**OD_{600} Control (4 h)**	**OD_{600} + Phage (4 h)**	**OD2/OD1 (4 h)**	**Effect at 4 h**	**OD_{600} Control (5 h)**	**OD_{600} + Phage (5 h)**	**OD2/OD1 (5 h)**	**Effect at 5 h**
SVP202	1	0.812	1.332	1.640	Resistant	0.737	1.567	2.126	Resistant
	2	0.812	1.683	2.073	Resistant	0.737	1.421	1.928	Resistant
	3	0.812	1.352	1.665	Resistant	0.737	1.862	2.526	Resistant
SVP204	1	0.436	0.945	2.167	Resistant	0.682	0.759	1.113	Resistant
	2	0.436	0.386	0.885	Susceptibility	0.682	0.572	0.839	Susceptibility
	3	0.436	0.573	1.314	Resistant	0.682	0.734	1.076	Resistant
SVP206	1	0.752	0.751	0.999	Susceptibility	0.682	0.701	1.028	Resistant
	2	0.752	0.812	1.080	Resistant	0.682	0.749	1.098	Resistant
	3	0.752	0.836	1.112	Resistant	0.682	0.752	1.103	Resistant
SVP208	1	0.588	0.489	0.832	Susceptibility	0.422	0.582	1.379	Resistant
	2	0.588	0.629	1.070	Resistant	0.422	0.635	1.505	Resistant
	3	0.588	0.682	1.160	Resistant	0.422	0.824	1.953	Resistant
SVP213	1	0.692	0.568	0.821	Susceptibility	0.386	0.435	1.127	Resistant
	2	0.692	0.738	1.066	Resistant	0.386	0.381	0.987	Susceptibility
	3	0.692	0.747	1.079	Resistant	0.386	0.527	1.365	Resistant

SVP217	1	0.452	0.553	1.223	Resistant	0.467	0.648	1.388	Resistant
	2	0.452	0.546	1.208	Resistant	0.467	0.683	1.463	Resistant
	3	0.452	0.684	1.513	Resistant	0.467	0.625	1.338	Resistant
SVP218	1	0.653	0.747	1.144	Resistant	0.573	0.469	0.818	Susceptibility
	2	0.653	0.838	1.283	Resistant	0.573	0.638	1.113	Resistant
	3	0.653	0.585	0.896	Susceptibility	0.573	0.889	1.551	Resistant
SVP223	1	0.463	0.568	1.227	Resistant	0.537	0.879	1.637	Resistant
	2	0.463	0.679	1.467	Resistant	0.537	0.893	1.663	Resistant
	3	0.463	0.711	1.536	Resistant	0.537	0.895	1.667	Resistant
SVP224	1	0.746	0.794	1.064	Resistant	0.853	0.946	1.109	Resistant
	2	0.746	0.785	1.052	Resistant	0.853	0.995	1.166	Resistant
	3	0.746	0.862	1.155	Resistant	0.853	0.886	1.039	Resistant
SVP225	1	0.482	0.754	1.564	Resistant	0.534	0.683	1.279	Resistant
	2	0.482	0.859	1.782	Resistant	0.534	0.583	1.092	Resistant
	3	0.482	0.611	1.268	Resistant	0.534	0.983	1.841	Resistant
SVP226	1	0.758	0.853	1.125	Resistant	0.587	0.812	1.383	Resistant
	2	0.758	0.889	1.173	Resistant	0.587	0.786	1.339	Resistant
	3	0.758	0.943	1.244	Resistant	0.587	0.898	1.530	Resistant
SVP227	1	0.642	0.715	1.114	Resistant	0.451	0.692	1.534	Resistant
	2	0.642	0.829	1.291	Resistant	0.451	0.554	1.228	Resistant
	3	0.642	0.754	1.174	Resistant	0.451	0.676	1.499	Resistant
SVP229	1	0.812	1.063	1.309	Resistant	0.683	0.872	1.277	Resistant
	2	0.812	1.145	1.410	Resistant	0.683	0.775	1.135	Resistant
	3	0.812	1.279	1.575	Resistant	0.683	0.891	1.305	Resistant
SVP230	1	0.482	0.303	0.629	Susceptibility	0.582	0.682	1.172	Resistant
	2	0.482	0.988	2.050	Resistant	0.582	0.427	0.734	Susceptibility
	3	0.482	0.886	1.838	Resistant	0.582	0.621	1.067	Resistant
SVP234	1	0.582	0.651	1.119	Resistant	0.797	0.983	1.233	Resistant
	2	0.582	0.739	1.270	Resistant	0.797	0.824	1.034	Resistant
	3	0.582	0.644	1.107	Resistant	0.797	0.993	1.246	Resistant
SVP240	1	0.422	0.584	1.384	Resistant	0.601	0.824	1.371	Resistant
	2	0.422	0.792	1.877	Resistant	0.601	0.768	1.278	Resistant
	3	0.422	0.681	1.614	Resistant	0.601	0.845	1.406	Resistant
