# Supplementary material for: Antimicrobial Resistance Profiles, Resistance Genes, and Their Association with Bacteriophage Susceptibility Among Vibrio parahaemolyticus Isolates from Zhanjiang Shrimp Aquaculture Farms
Source: Microorganisms. 2026 Sep 9;14(9):2004. doi: 10.3390/microorganisms14092004 (PMC13608986; doi:10.3390/microorganisms14092004)
Supplement: Supplementary file 1 [file microorganisms-14-02004-s001.zip › Supplementary Table S5.pdf]

Supplementary Table S5. Primer sequences, target genes, and expected amplicon sizes used for PCR detection of antimicrobial resistance and phage-host interaction-associated genes in *Vibrio parahaemolyticus* isolates.

**Gene**	**Forward primer (5′–3′)**	**Reverse primer (5′–3′)**	**Amplicon size (bp)**
tetA	CCTGATGGCGGTCTTCTTCA	CACCTGCCTGGACAACATTG	343
tetB	TTATCATGCCAGTCTTGCCAAC	CTAACCAACCGAACCACTTCAC	342
blaTEM	GCCAACTTACTTCTGACAACGA	ATCCGCCTCCATCCAGTCT	240
blaCTX-M	AAGCCTGCCGATCTGGTTAA	GGTATTCAGCGTAGGTTCAGTG	222
blaOXA	AGCCGTCAATGGTGTCTTCT	GCATTCTTACTTCGCCAACTTC	293
qnrA	TATCAGTGTGACTTCAGCCATT	TGAGATATAAGCCGAGCAGAAG	276
Sul1	CGATATTGCTGAGGCGGACT	AACGCCGACTTCAGCTTTTG	264
aphA	GCTGGTTACTTGCGTACTAGAA	AGTTGAAGGCGTTGCGTAG	274
luxS	GGACCACACTCGTATGAATGC	TGCGACTTGCTGCTCTGAT	277
rfb	AAGGCTGCTTGATTACGATTGA	ACATCTTGCTCTGTTGCTACTG	340
hsdM	GGTGATGCCTACGAGTTCCT	AGTTGTAAGTGGTGTGGTTGAG	253
Ugd	ACCGTGTTATTGGTGGTCTTAC	TCGTGCCGTATGAATAATCTGG	347
