# Supplementary material for: Antimicrobial Resistance Profiles, Resistance Genes, and Their Association with Bacteriophage Susceptibility Among Vibrio parahaemolyticus Isolates from Zhanjiang Shrimp Aquaculture Farms
Source: Microorganisms. 2026 Sep 9;14(9):2004. doi: 10.3390/microorganisms14092004 (PMC13608986; doi:10.3390/microorganisms14092004)
Supplement: Supplementary file 1 [file microorganisms-14-02004-s001.zip › Supplementary Table S7.pdf]

Supplementary Table S7. Site-specific distribution of phage-resistant and phage-susceptible *Vibrio parahaemolyticus* isolates at 4 h and 5 h incubation periods.

Sampling site	Total isolates	Resistant (4 h)	Susceptible (4 h)	Resistant (5 h)	Susceptible (5 h)
Huguang Town (HVP)	20	19	1	19	1
Lindong Village (LVP)	20	7	13	5	15
Wensheng Village (WVP)	20	11	9	13	7
Xinliao Town (XVP)	20	9	11	10	10
Yingzai Town (YVP)	20	20	0	20	0
Gaoliao Village (GVP)	16	11	5	7	9
Donghai Dao (Shrimp) (SVP)	16	16	0	16	0
**Total**	**132**	**93**	**39**	**90**	**42**

Each isolate was tested using three biological replicates. The final isolate phenotype was assigned based on the majority response among replicates. Isolates with $OD_2/OD_1 \geq 1$ were classified as resistant, whereas isolates with $OD_2/OD_1 < 1$ were classified as susceptible (Kutter et al., 2010; Merabishvili et al., 2009).
